# Structural Descriptors for Subunit Interface Regions in Homodimers: Effect of Lipid Membrane and Secondary Structure Type

**DOI:** 10.1021/acs.jcim.4c01233

**Published:** 2025-03-27

**Authors:** Aslı Yüksek, Batuhan Yıkınç, İrem Nayır, Defne Alnıgeniş, Vahap Gazi Fidan, Tayyip Topuz, Ebru Demet Akten

**Affiliations:** † Department of Molecular Biology and Genetics, Faculty of Engineering and Natural Sciences, Kadir Has University, 34083 Fatih, Istanbul, Turkey; ‡ Ph.D. Program of Computer Engineering, School of Graduate Studies, Kadir Has University, 34083 Fatih, Istanbul, Turkey

## Abstract

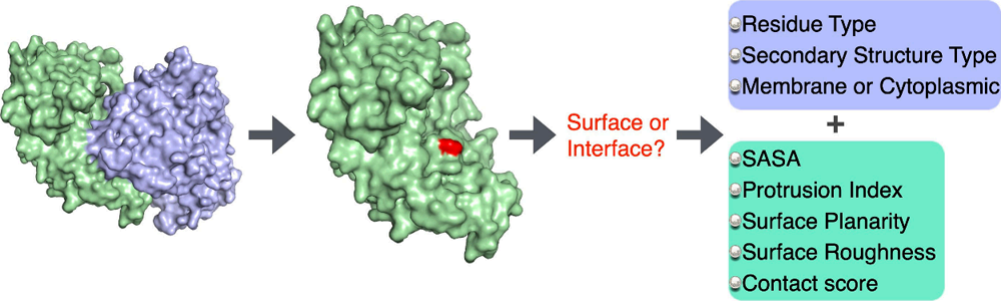

A total of 1311 homodimers were collected and analyzed in three different categories to highlight the impact of lipid environment and secondary structure type: 422 cytoplasmic α-helix, 411 cytoplasmic β-strand, and 478 membrane complexes. Structural features of the interface connecting two monomers were investigated and compared to those of the non-interface surface. Every residue on the surface of each monomer was explored based on four attributes: solvent-accessible surface area (SASA), protrusion index (C_*x*_), surface planarity, and surface roughness. SASA and C_*x*_ distribution profiles clearly distinguished the interface from the surface in all categories, where the rim of the interface displayed higher SASA and C_*x*_ values than the rest of the surface. Surface residues in membrane complexes protruded less than cytoplasmic ones due to the hydrophobic environment, and consequently, the difference between surface and interface residues became less noticeable in that category. Cytoplasmic β-strand complexes displayed markedly lower SASA at the interface core than at the surface. The major distinction between the surface and interface was achieved through surface roughness, which displayed significantly higher values for the interface than the surface, especially in cytoplasmic complexes. Clearly, a surface which is relatively rugged favors the association of two monomers through multiple van der Waals interactions and hydrogen-bond formations. Another structural descriptor with strong distinguishing ability was surface planarity, which was higher at the interface than at the non-interface surface. Surface flatness would eventually facilitate the interconnectedness of an interface with a network of residue pairs bridging two complementary surfaces. Analysis of contact pairs revealed that hydrophobic pairs have the highest frequency of occurrence in the lipid environment of membrane complexes. However, despite the scarcity of polar residues at the interface, the likelihood of observing a contact between polar residues was markedly higher than that of hydrophobic ones.

## Introduction

Identification of subunit interaction sites is critical for understanding the fundamental nature of protein complexes, their function, and ultimately the mechanism of several diseases. Complex structures require both geometric and chemical complementarity between monomeric units, which are crucial for determining the specific interactions connecting these subunits.^1−3^ Several studies attempted to identify chemical and physical characteristics that would differentiate the interaction site, namely, the interface, from the non-interface surface regions. Previously, interaction sites have been determined as hydrophobic, planar, globular, and protruding.^4−6^ However, the database used in those studies was restricted to a few protein complexes. Analysis of protein–protein interaction sites using surface patches conducted by Jones and Thornton^4^ was performed using 28 homodimers, 20 heterodimers, and a few antibody–antigen complexes. A relatively more recent study conducted by Glaser et al.^7^ used a nonredundant set of 621 protein complexes. In our study, we doubled that number, using a total of 1311 homodimers. This extensive amount of crystallographic data extracted from the Protein Data Bank (PDB)^8^ was analyzed using surface patches adopted by Jones and Thornton. Each dimer complex was decomposed into its monomeric units, and for each monomeric surface, the distribution of four structural features was determined; solvent-accessible surface area (SASA), protrusion index (C_*x*_), surface roughness, and surface planarity. Accordingly, surface roughness and surface planarity were identified as the two most powerful descriptors to distinguish interface from non-interface surface residues. The interface was found to be planar, and the roughness was observed to be higher at the interface. These findings emphasized the structural features of the interface, which displays a rough but overall flat terrain as opposed to a smoother but wavy non-interface surface.

In addition, the effect of the lipid environment and secondary structure type constituting the interface was investigated. For that purpose, protein complexes were categorized based on their local environment and also based on the secondary structure type of the interface. Clearly, the lipid environment had a significant degree of impact on the residue composition, residue propensity, and frequency of residue pairs in contact bridging the two monomers. This type of categorization will be the first in the literature, as previous studies focused on a single database that included complexes of varying structural features and thus totally ignored the uniqueness of each category. Another important structural descriptor to be used for the final construction of the dimer from its subunits is the type of residues that would likely interact when two subunits come in close contact. This type of information would be used for the final assembly of the complex after the identification of potential interface surface patches.

## Methods

### Dataset Preparation

**Figure 1:**
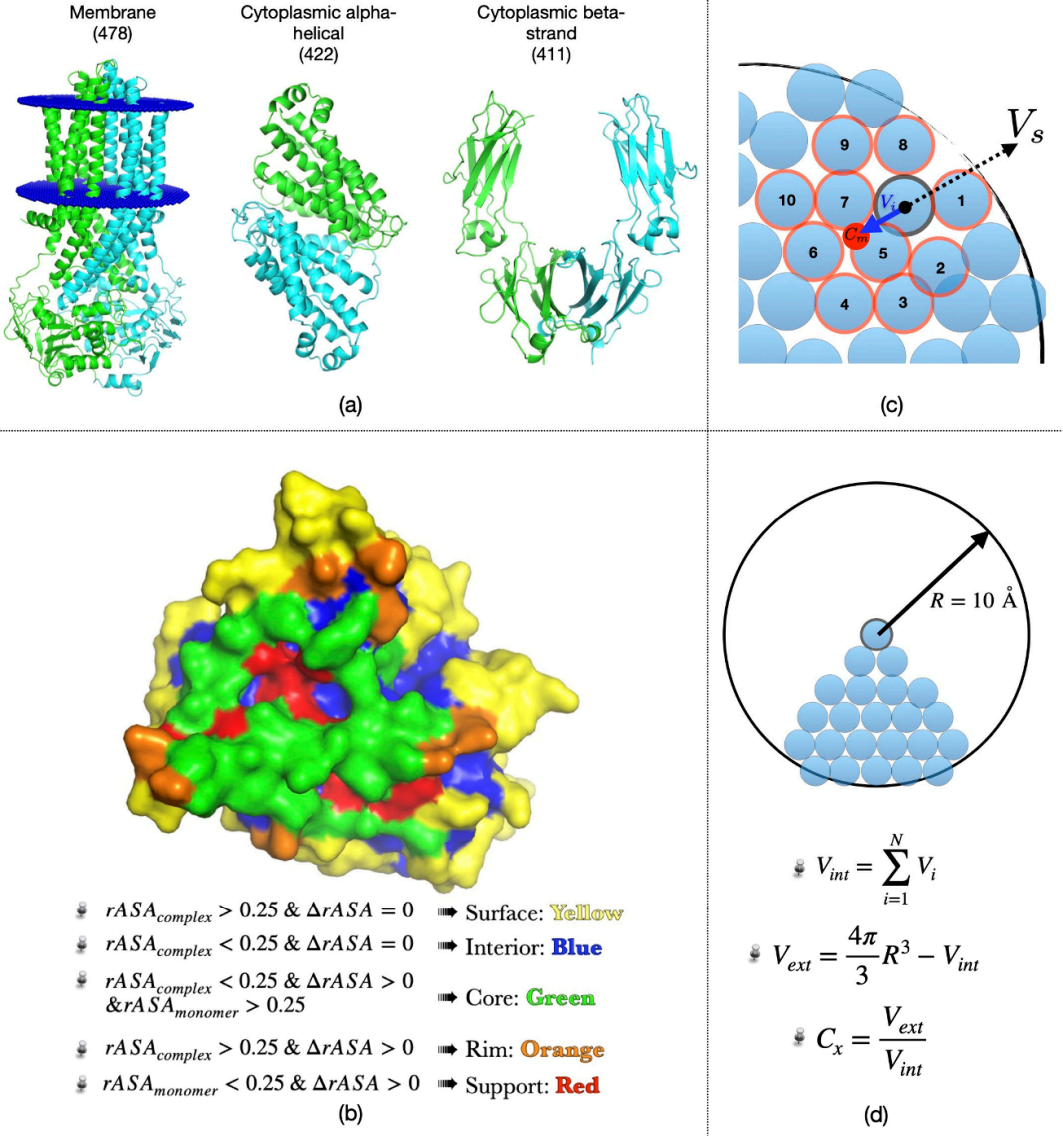
(a) Representative protein structure taken from each category established based on the secondary structure type of the interface and the surrounding environment. (b) Location of five different regions in the complex represented on one monomer only with an open interface. (c, d) Illustrations of (c) surface patch and (d) protrusion index calculations. See the text for details.

A total of 1311 protein homodimers were selected from the Protein Data Bank^8^ after an elaborate filtering protocol. They were analyzed in three categories (Figure 1a): cytoplasmic alpha (422), cytoplasmic beta (411), and membrane (478). Here “alpha” and “beta” indicate the type of secondary structure (α-helical/β-strand) present at the interface. As the majority of membrane proteins incorporated α-helices, the effect of the secondary structure type was investigated using cytoplasmic proteins only. Membrane proteins were extracted from the OPM database,^9^ which provides the same structural information as in the PDB and an additional entry for the predicted position of inner/outer membrane layers which is critical in this study to differentiate the membrane’s hydrophobic interior from its aqueous exterior. No peripheral membrane proteins were included in the database.

Our goal was to assemble structures as unique as possible; thus, PDB was searched among seven different enzyme categories: oxidoreductase, transferase, hydrolase, lyase, isomerase, ligase, and translocase. This yielded 2500 cytoplasmic alpha and 2500 cytoplasmic beta structures. A considerable number were initially discarded, as they provided an artifically constructed dimer instead of a biological assembly. Another basis for exclusion was the absence of a compact interface. The lowest numbers of residues buried at the interface of cytoplasmic alpha, cytoplasmic beta, and membrane complexes were determined as 14, 17, and 18, respectively, considered as the minimum numbers of buried residues required for a tightly packed interface. Following this detailed examination for structural consistency, the number in each category was reduced to nearly 800 structures. For membrane proteins, all 1100 dimer complexes deposited in the OPM database were examined for structural consistency. Out of 1100 complexes, a total of 700 were selected.

Raw data files collected from databanks needed additional corrections, as some residues had either two entries in the coordinate file or no entries at all. Also, some reformatting was necessary for residue ID columns: e.g., in cases where the total number of residues exceeded 999, the ID became 000 due to lack of space; also some negative residue IDs appeared. Missing residues in the structure were completed using homology modeling tool MODELLER,^10,11^ taking the complete primary sequence and remapping it onto the original structure used as a template.

Many entries in the database represent the same protein, either complexed with different ligands or mutated. To avoid redundancy, all collected structures were sequentially aligned using Clustal Omega,^12^ with 85% as the selected threshold for maximum identity. Only one representative structure was arbitrarily selected among *N* proteins with a sequence identity ≥85%. Accordingly, nearly half of the collected data in each category were discarded, and a total of 1311 homodimers (478 membrane, 422 cytoplasmic α-helical, and 411 cytoplasmic β-strand) were left for analysis. The final refinement was to remove any cofactor, ligand, and/or water from the structure.

### Solvent-Accessible Surface Area to Determine Residue Locations

Each dimer complex was divided into five different locations (Figure 1b): *interior*, *surface*, *core*, *rim*, and *support*. Each residue was assigned to one of these regions based on its SASA value in the protein complex. SASA was determined via Visual Molecular Dynamics (VMD),^13^ which applies the Shrake and Rupley algorithm^14^ with a probe radius of 1.4 Å. Residue location was then determined based on relative SASA (denoted as rASA) following the generalized definition by Levy.^15^ rASA was determined by dividing the SASA by the maximum theoretical SASA of that residue X observed in a tripeptide configuration Gly–X–Gly.^16^ Accordingly, a residue was considered to be buried in the interior when its rASA was below a cutoff value of 0.25. As noted in Figure 1b, residues at the interface were identified as those displaying a change in their rASA when going from the monomeric state to the complex state, i.e., ΔrASA ≠ 0. In Levy’s description, the interface was further decomposed into support, rim, and core.

### Propensity of Each Residue for Different Locations

Four propensity values were evaluated based on SASA of each residue type at different locations. Each dimer was decomposed into its monomeric units, and SASA values were determined in the monomeric state. The surface region excludes all interface residues. The propensity value *P*(*i*_*k*/*m*_) represents how much the frequency of SASA of residue *i* at a specific location *k* is different from that at another location *m*:
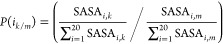
1To emphasize the difference between non-interface surface and interface, the propensity was determined for the interface core and rim with respect to non-interface surface. Another component of the interface that is situated in the interior of the monomer is the support. The propensity for the support region (*k*) was evaluated against the interior (*m*). Thus, four different *P*(*i*_*k*/*m*_) were evaluated: interface (*k*)/surface (*m*), rim (*k*)/surface (*m*), core (*k*)/surface (*m*), and support (*k*)/interior (*m*).

### Surface Patch Defined for Each Surface Residue on the Monomer

Adapted from the work by Jones and Thornton,^19^ the patch was defined as a central surface residue with its *n* nearest surface neighbors based on proximity of their C^α^ atoms. The algorithm can be divided into two stages. In the first stage, the center of mass *C*_m_ was calculated for each residue with its 10 nearest neighbors, as depicted in Figure 1c. A solvent vector, *V*_s_, was then defined as the opposite vector of *V*_*i*_, which points from the central residue to *C*_m_. Thus, a vector pointing to the solvent was assigned to each surface residue. In the second stage, a surface residue was selected randomly as the center of the newly created patch. Then a neighboring residue *i* was included in that surface patch of size *n* if the angle between its solvent vector *V*_s_ and that of the central residue of the patch was less than 110°. In this study, 3, 9, 15, 17, and 20 were used as different patch sizes. This procedure eventually produced several contiguous, overlapping patches.

### Protrusion Index for Surface Residues

Using the definition by Pintar et al.,^17^ the protrusion index (*C*_*x*_) of each surface atom was determined using a sphere of 10 Å radius centered around each non-hydrogen atom, as depicted in Figure 1d. The free volume inside the sphere (*V*_ext_) was divided by the total volume occupied by the atoms (*V*_int_) to obtain *C*_*x*_ (=*V*_ext_/*V*_int_). Instead of Pintar’s definition of fixed atomic volume at 20.1 Å^3^, each atom was assigned to a unique volume taken as the average value determined for 64 protein structures using the classical Voronoi procedure by Pontius et al.^18^ The protrusion index of a residue was taken as the mean *C*_*x*_ of all atoms in that residue.

### Surface Roughness

Adapting the definition of Lewis and Rees,^20^ surface roughness was defined as *∂* log SASA/*∂* log *R*, which is the change in SASA as a function of radius *R* of the rolling spherical probe atom. The higher the irregularity on the surface, the larger the change will become. The radius *R* was increased from 0.2 Å to 4.0 Å with a step size of 0.1 Å.

### Surface Planarity

The planarity of each surface residue was determined as the root-mean-square deviation (rmsd) of all atoms in the patch defined for that residue from the least-squares fit plane created by all atoms in the patch. Thus, a low rmsd was interpreted as a planar patch.

### Observed Contact Frequency at the interface

In a dimer complex with monomers A and B, the observed contact frequency *C*_*i*, *j*_ of a residue pair of type *ij* at the interface was determined as the number of residues of type *i* in monomer A in contact with residues of type *j* in monomer B (*N*_*i*A,*j*B_) plus the number of residues of type *i* in monomer B in contact with residues of type *j* in monomer A (*N*_*j*A,*i*B_) divided by the total number of contacts at the interface:
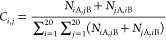
2Distance thresholds were taken as 6, 5, and 4 Å for backbone–backbone, backbone–side chain, and side chain–side chain contacts, respectively. To be considered in contact, either one of these distance criteria must be satisfied. A score value *S*_*i*, *j*_ was then determined by taking the ratio of the observed frequency to the expected frequency:
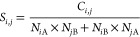
3

## Results and Discussion

**Figure 2:**
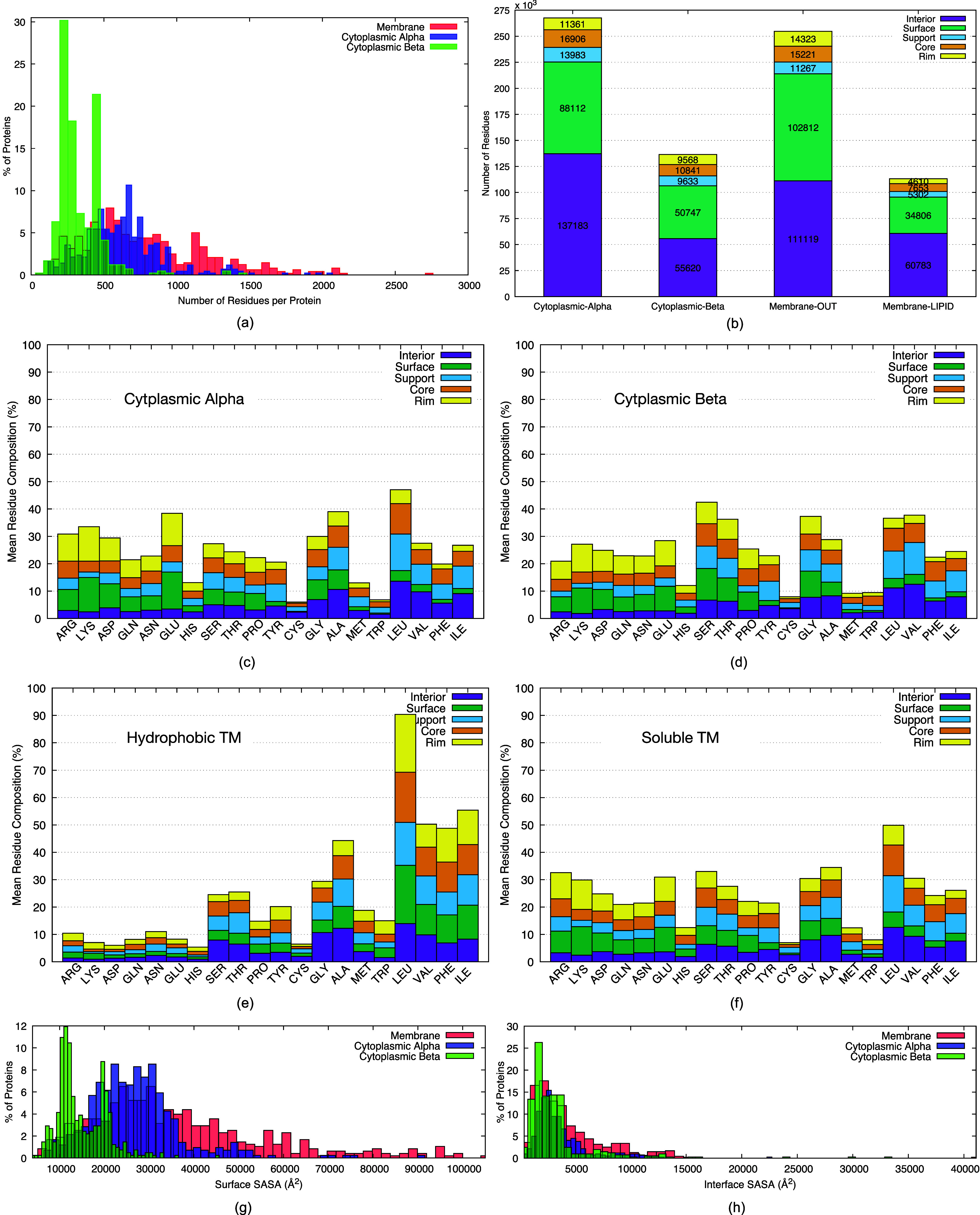
(a, b) Distribution profiles of (a) protein complexes in each category and (b) numbers of residues in different parts of the complex. (c–f) Mean residue composition in different regions of the protein for (c) cytoplasmic alpha, (d) cytoplasmic beta, (e) hydrophobic TM, and (f) soluble TM. (g, h) Distribution profiles of SASA values of (g) exposed surface and (h) buried interface in the complex. The interface area should be divided by ∼2 to get the area corresponding to one monomer.

Three different categories of homodimers were analyzed to distinguish their structural features that will be used in a machine learning model for interface prediction in the succeeding study (Figure 2a). Membrane proteins were further categorized as “hydrophobic TM” (where TM = transmembrane) for protein parts embedded inside the lipid bilayer and “soluble TM” for parts extending toward either cytoplasmic or extracellular parts, since the environment plays a key role for protein–protein interactions.^21^ The widest range of protein sizes was observed in membrane proteins, where the number of residues changed between 60 and 2733, whereas cytoplasmic categories incorporated moderate-size proteins; those with α-helices displayed a wider distribution [136–2050] than those with β-strands [58–1454]. For each category, the total number of residues in five different regions was determined, and the results are shown in Figure 2b. Accordingly, the largest amount of data belonged to α-helical cytoplasmic proteins, with a total of 267,543 residues, half of which were buried in the interior. The lowest amount of packing was observed in the cytoplasmic beta category, with 40.1% of residues located in the interior, whereas the highest degree of packing was encountered in hydrophobic TM, with a 53.7% contribution.

Proteins were further decomposed into their repeating units, and the mean residue compositions (%mrc) in each of the five locations are depicted in Figure 2c–f, where the residues on the *x* axis are listed from left to right based on their Eisenberg normalized hydrophobicity constants.^22^ In both cytoplasmic alpha and beta complexes, polar residues were distributed mainly on the surface and the interface rim, whereas hydrophobic residues dominated the interior and the interface support. In the interface core, the %mrc of hydrophobic residues was slightly higher than that of polar ones. Overall, moderate differences existed between cytoplasmic alpha and beta categories, where the amount of polar residues was slightly lower in cytoplasmic beta than in cytoplasmic alpha. Also, higher %mrc values for two residues with hydroxylic side chains, Ser and Thr, were observed on the surface and the interface of cytoplasmic beta complexes; Ser had 42.5% composition in total, which was the highest among all residues, followed by Val, Gly, Leu, and Thr with 36–37%. Moreover, the difference between cytoplasmic and membrane complexes was distinct (Figure 2e,f). First, there was the scarcity of polar residues everywhere in the lipid side of the membrane proteins. Also, the high composition of hydrophobic residues, especially Leu, was observed throughout the structure, especially on the surface and the interface. The resemblance in %mrc between cytoplasmic alpha and the aqueous part of membrane proteins was expected, as the membrane’s outer edge experiences the same polar environment as cytoplasmic proteins.

SASA, as one of the key structural descriptors for distinguishing surface from interface, was identified for each protein. Distribution profiles were determined for surface area of the complex and the one buried at the interface between two monomeric subunits, as illustrated in Figure 2g,h, respectively. Buried surface area (BSA) was simply defined as the sum of the SASA of two disconnected subunits minus that of the complex. As indicated in Miller’s work,^23^ the surface area of the complex was greatly determined by the size, and thus, there is a close correspondence between SASA illustrated in Figure 2g and the number of residues displayed in Figure 2a. BSA was particularly similar in all three categories, exhibiting values between 400 and 15,000 Å^2^ with a mean BSA around 2500 Å^2^. This is in accordance with the minimum interface size of 600 Å^2^ that was proposed by Jones and Thornton^4^ and by Bogan and Thorn^24^ based on their comprehensive survey of reversible protein–protein complexes. This indicates that although the complex sizes may vary significantly, BSA was preserved.

According to the empirical correlation between SASA of an amino acid and its free energy of transfer to a water solution, 1 Å^2^ of surface area corresponds to 25 cal mol^–1^ of hydrophobic free energy.^25^ Thus, the hydrophobic contribution to bury nearly 2500 Å^2^ of surface area upon complexation would amount to ∼60 kcal/mol. This contribution would be intensified if the buried residues consisted of hydrophobic residues in a polar cytoplasmic environment. This was clearly reflected in the propensity values for cytoplasmic alpha and beta complexes depicted in Supplementary Figure 1, where the frequency of hydrophobic residues at the core region was up to 7 times bigger than at the surface. The difference was especially pronounced for Tyr, Trp, and Phe, which are the only three amino acids with an aromatic ring in their side chains, in addition to Cys. Thus, the stability of the interface in cytoplasmic homodimers was mainly established via aromatic rings or disulfide bridges. For membrane proteins, the transfer of hydrophobic residues to the interface would not be as favorable as for cytoplasmic proteins since the membrane environment is already hydrophobic. This expectation was clearly reflected in the propensity values of the hydrophobic residues. On the other hand, the transfer of polar residues to the interface might be more favorable, as they would encounter other polar residues at the interface more frequently than at the surface, which is in direct contact with the hydrophobic lipid bilayer. Once more, this expectation was validated with propensity values above 1 for polar residues (see Supplementary Figure 1). An important finding was observed for hydrophobic residues inside the lipid bilayer, which displayed a preference for the support interface over the interior. On the other hand, polar residues in all protein categories tend to prefer to be buried deep inside the interior than to be at the support.

**Figure 3:**
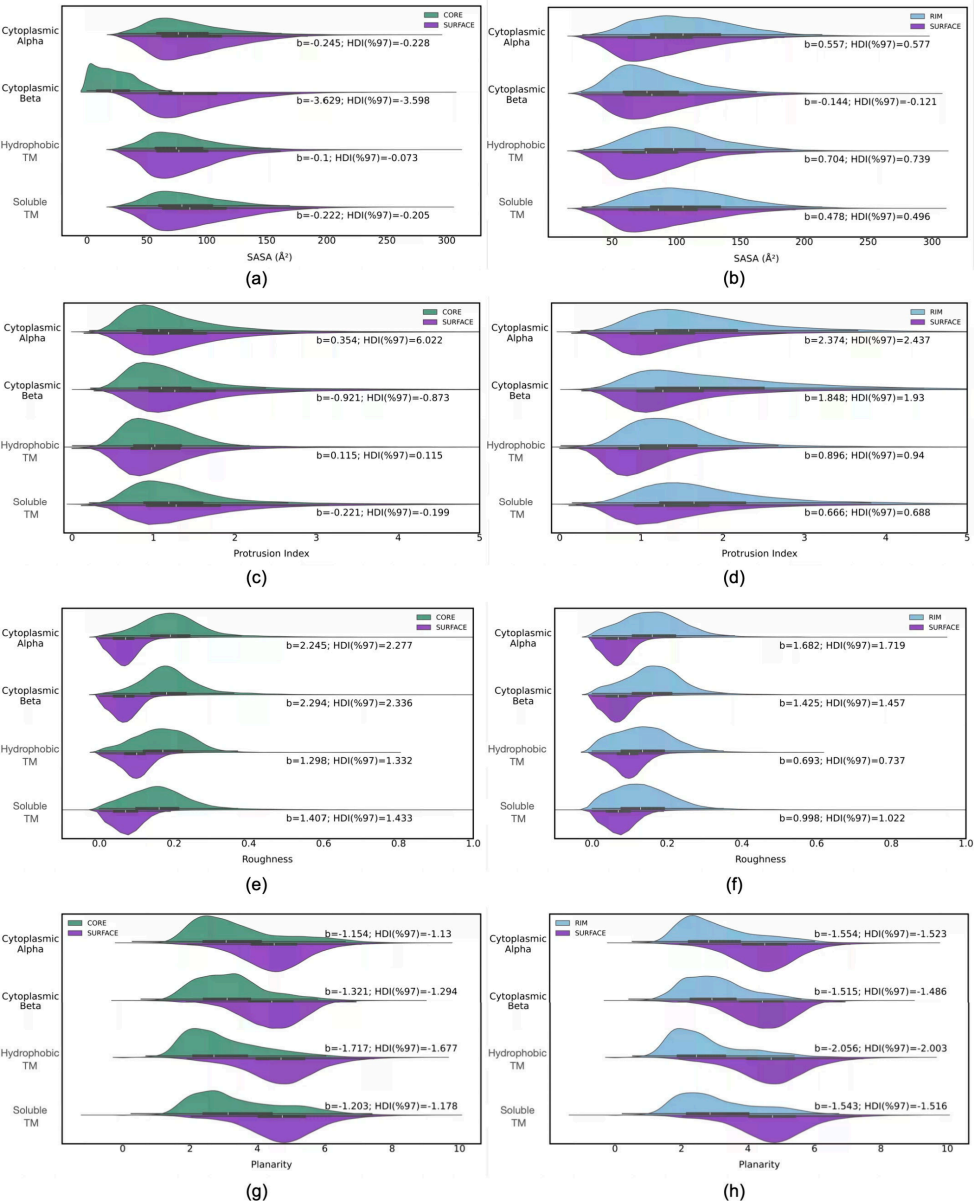
Distribution profiles of (a, b) SASA, (c, d) protrusion index, (e, f) roughness, and (g, h) planarity values of all residues at the surface region with those at the core and the rim in four different categories. Effect size *b* and high-density probability interval HDI (97%) values are noted.

To resolve the distinction between the surface and interface, the distribution of SASA for all residues at the surface was illustrated with those at the core and rim of the interface in Figure 3 using split-violin representations. To further evaluate the significance of their differences, a full Bayesian parameter estimation was performed on every pair of distributions using a Markov chain Monte Carlo algorithm via PyMC3.^26^ Two parameters, the effect size *b*, defined as the difference in means scaled by the pooled estimate of standard deviation,
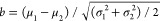
4and a 97% credible interval, the high-density probability interval (HDI) around the effect size, were calculated. The farther the effect size and 97% HDI are from 0, the more significant the difference in SASA distributions between two regions is. Unlike a single-point value of *p* ≤ 0.05 in the standard *t* test, Bayesian estimation uses an entire distribution of parameters for calculating the effect size.

As mentioned in Methods, SASA values at the core and the rim were determined in each monomeric unit after the complex was disassembled. Except for cytoplasmic beta, SASA distributions in both surface and core regions were similar, with effect sizes of −0.24,–0.1, and–0.22 for cytoplasmic alpha, hydrophobic TM, and soluble TM categories, respectively (see Figure 3a). Negative values of the effect size indicated a slightly lower mean SASA at the core. For cytoplasmic beta, the SASAs of the core residues were significantly lower than those at the surface, with an effect size of −3.63, which makes SASA a strong discriminator between surface and interface. The distinction between interface rim and surface was the weakest in cytoplasmic beta complexes, with an effect size of −0.14 (see Figure 3b). For membrane lipids, a distinct SASA profile for the rim and surface with an effect size of 0.70 indicated a higher mean SASA at the rim. Similarly, distribution profiles of SASA in cytoplasmic alpha and soluble TM indicated a higher mean of SASA for the rim than for the surface, with effect sizes of 0.56 and 0.48, respectively.

It is important to deconstruct SASA into residue types to uncover the discriminating power of each residue separately. Martins et al.^27^ highlighted the importance of residue standardization in improving the recognition of hot spot regions which account for most of the binding energy of two proteins. As listed in Supplementary Table 2, all 20 residues grouped as polar and hydrophobic based on their hydrophobicity constants^28^ displayed different distribution profiles in surface and interface rim associated with distinct effect sizes. For cytoplasmic alpha, residues displaying higher mean values at the rim as opposed to surface were mostly hydrophobic, especially Leu, Phe, and Trp. Thus, any one of these three residues’ SASA would be a strong descriptor for locating an interface. Similarly, for the lipid part of the membrane, SASA of polar Asn and Glu and hydrophobic Tyr, Cys, and Met can be effectively used to distinguish a rim from a surface.

Additionally, the SASA distribution profiles of all 20 residues are presented in Supplementary Figure 2 using box-and-whisker plots. For all residue types in all three categories (cytoplasmic alpha, hydrophobic TM, and soluble TM), SASAs at the rim were significantly higher than those at either core or surface. In cytoplasmic beta, all residues had slightly higher SASAs at the surface than at the rim (Supplementary Table 2). The rim represents the outer edge of the interface, and thus, any residue type irrespective of its size, shape, and chemical feature would display a higher degree of solvent exposure when located at the edge of an interface as opposed to the core. Moreover, the stability of an interface between two monomers likely requires a protuberance on the surface of the protein, which will manifested by higher SASA values, similar to the confines of a secure lock.

Considering the outer edge of the interface, we expected to observe the rim as the most solvent-accessible region in the complex. For cytoplasmic alpha, when the surface was compared to the interface core, SASA values of polar residues were slightly higher at the surface than those at the core. A nearly opposite trend was observed for hydrophobic residues such as Trp, Leu, and Phe, which displayed slightly higher SASAs at the core than at the surface. For hydrophobic TM, difference between surface and core becomes negligible, yet the rim can be located using the two polar residues Asn and Glu, aromatic Tyr, and sulfur-containing Cys and Met (see Supplementary Tables 2 and 3). For cytoplasmic beta, the major distinguishing feature between surface and interface was SASA, which was exceptionally higher for surface residues than those at the core.

Another important structural descriptor was the protrusion index, which measures how much a residue protrudes from the surface. It usually varies between 0 and 10, yet most *C*_*x*_ values were observed betwen 0.5 and 2.5 (see Figure 3c,d). This range also coincides with those determined by Pintar et al.^17^ A threshold of 2 would indicate a free volume twice as large as the occupied volume, which would represent a protruding residue. The difference in distribution profiles between surface and rim was significant, with effect sizes of 2.37 and 1.85 for cytoplasmic alpha and beta complexes, respectively. For all categories, the distribution profiles were indistinguishable for surface and core, with effect sizes close to 0 and mean C_*x*_ varying between 1.00 and 1.25. On the other hand, the distinction between rim and surface was noticeable. In addition to distribution profiles for each residue type, Supplementary Figure 3 also provides the corresponding percentages of residues with C_*x*_ values above the threshold value of 2. Especially for cytoplasmic beta, C_*x*_ was highly discriminating between rim and surface regions due to the distinct conformational arrangement of β-strands as opposed to the more compact α-helical arrangements. In cytoplasmic alpha, one-third of residues displayed C_*x*_ greater than or equal to 2 whereas in cytoplasmic beta, nearly half of the residues were protruding. For membrane categories, rim residues embedded inside the lipid bilayer protruded less than those in the other three categories due to the membrane’s hydrophobic environment. Also, the distribution range in the lipid membrane was significantly narrower than either cytoplasmic category or soluble TMs. The highest C_*x*_ values were observed among rim residues throughout all four categories, yet they were more pronounced in cytoplasmic beta complexes and for Cys, Gly, Ala, and Met.

Surface roughness and surface planarity were identified as the strongest structural descriptors (Figures 3e–h). The roughness of the non-interface surface was significantly lower than those of the core and the rim. High roughness was particularly visible in the core region. The distinction in distribution profiles between surface and core was significant in cytoplasmic alpha and beta categories with effect sizes of 2.24 and 2.29, respectively. These results indicated a smooth, uniform characteristic for the surface. On the other hand, an irregular, bumpy interface appeared, as this would enable more connection sites between two interacting monomers for increased stability. As also stated by Pettit and Bowie,^29^ rough surfaces allow more van der Waals (vdW) contacts which can fit into a small volume, and those contacts are essential for the stability of the complex. The study of Wu et al.^30,31^ conducted on 1190 dimers identified interface regions to be rough compared to the surface. Our results indicated the degree of roughness, *∂* log SASA/*∂* log *R*, to be the highest at the core and nearly 3 times higher than those at the surface.

Surface planarity was defined as the root-mean-square distance of all atoms in the patch from the least-squares plane; thus, the lower the rmsd value, the greater the planarity is. As depicted in Figure 3g,h, surface residues had lower surface planarity (higher rmsd) than those at the interface. Interestingly, the difference in surface planarity was especially pronounced in the lipid regions of the membrane complexes. According to the review conducted by Jones and Thornton,^4^ average planarity was determined as 3.5 ± 1.7 Å for homodimers, which coincided with our planarity values of core residues. Nearly half of the 28 homodimers analyzed by Jones and Thornton displayed high planarity at the interface, while other interfaces were relatively nonplanar. In our study, for a large database composed of 1311 homodimers, the distribution profiles indicated a mean rmsd of 5 Å for non-interface surface and 3 Å for interface, i.e., the average planarity was increased nearly 50% at the interface. Comparison of distribution profiles between surface and interface (core/rim) displayed the significance of their differences, with effect sizes between −1.72 and −1.15. The interface lodged inside the membrane displayed the highest difference in distribution profile from that observed at the surface, with an effect size of −1.72.

Figure 3e–h illustrate the roughness and planarity for a patch size of 20 Å. Similar results were obtained for patch sizes of 3, 9, 15, and 17 Å (see Supplementary Figures 4 and 5). However, the highest difference between interface and non-interface surface was observed for the patch size of 20 Å. In addition, per-residue roughness and planarity were determined (Supplementary Figures 6 and 7). No major difference in their distribution was observed among residue types.

The final step of the analysis consists of identifying the percent fraction of interface residues in one monomer that interacted with those in the second monomer, as displayed in Supplementary Figure 8. Accordingly, in cytoplasmic alpha complexes, 1.7% of contacts consisted of Leu-Ala and Leu-Leu pairs. In fact, Leu was the major contributor, as it was observed in 18% of all contacts. Also, basic Arg was observed to interact with acidic Glu in 1.3% of the contacts. In cytoplasmic beta complexes, Leu was still the major contributor with 14% frequency of occurrence, yet Ser, Phe, and Gly were detected in contact pairs as frequently as Leu. In fact, Leu-Phe constituted 1.6% of all contacts. Membrane complexes displayed different frequency profiles in both lipid and aqueous environments. In the lipid phase, Leu was the leading residue with 27% frequency of occurrence in contact pairs. Contact occurred mostly among hydrophobic residues, which also incorporated Ser and Thr. Soluble TM displayed a profile similar to that of cytoplasmic alpha with Leu as the most frequently observed residue in contact pairs.

**Figure 4:**
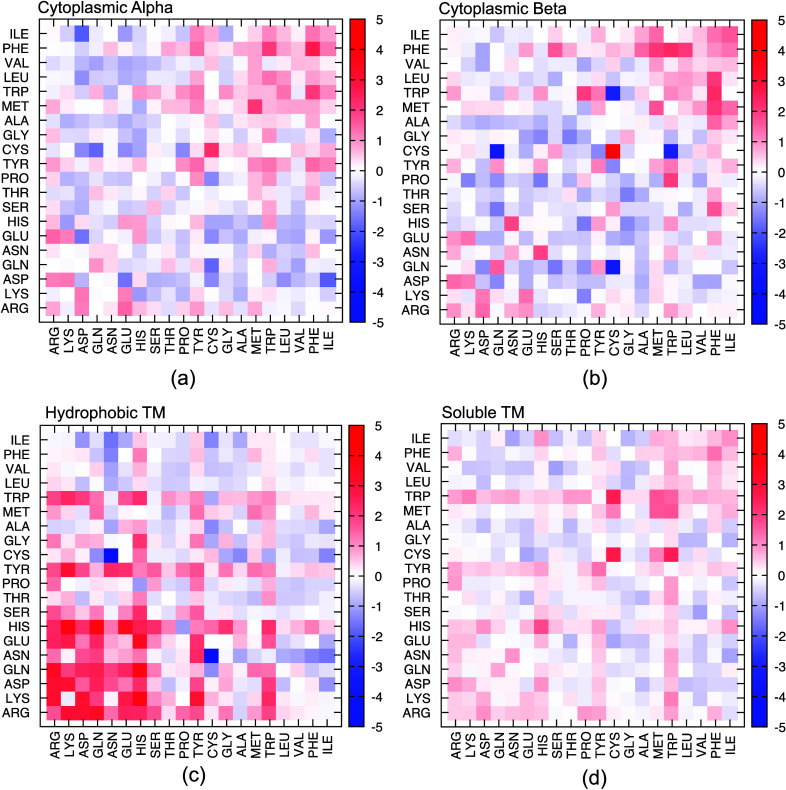
Likelihood of contacts between residues from two monomers at the interface. See Methods for details.

Next, the likelihood of contacts at the interface was determined (Figure 4). Although contact between polar residues was rarely observed in all four categories, the likelihood of a contact between two polar residues *S*_*i*, *j*_ when they appeared at the interface was significantly higher than a random occurrence, especially when that interface was located inside the lipid bilayer. Moreover, although the frequency of hydrophobic–hydrophobic contacts was prevalent at the interface for membrane lipid, the chance of observing hydrophobic–hydrophobic contact was random (*S*_*i*, *j*_ ∼ 0), whereas it was nearly twice the random occurrence in cytoplasmic alpha and nearly tripled in beta protein complexes (see top right corner in Figure 4b).

In all categories, polar–polar contacts were significantly rare, especially in the lipid interface of membrane proteins (see Supplementary Figure 8c), yet the likelihood of observing a pair of polar residues in contact at the interface was 3 to 4 times higher than a random occurrence, as depicted in Figure 4c. This suggests that when two polar residues are present at the interface of a complex in the lipid side, they will be most likely positioned in proximity so that they can create a contact pair. Furthermore, in membrane proteins, in addition to polar residues, Trp or Tyr was equally expected to be observed in a contact pair with any other polar residue in case they were located at the interface. This result was consistent with the work of Bogan and Thorn,^24^ which identified Trp, Tyr, and Arg as the three common residues to be part of the hot spots at the interface that contributed most to the free energy of binding of two monomers.

## Concluding Remarks

Homodimer complexes were investigated based on residue composition, SASA, protrusion index, surface roughness, surface planarity, and residue–residue contact preferences at the interface. Our dataset composed of 1311 homodimers with 771,764 residues was significantly larger than those used in previous studies. The dataset was categorized into three groups: 422 cytoplasmic alpha, 411 cytoplasmic beta, and 478 membrane complexes. Accordingly the effect of lipid environment and secondary structure type were highlighted for the first time in the literature. The effect of the lipid environment was noticeable in mean residue compositions. Hydrophobic residues, especially Leu, were everywhere in membrane proteins, whereas in cytoplasmic proteins they were mainly observed at the interior. Polar residues were rarely detected in membrane proteins, yet they were abundant on the surface and interface rim.

The propensity value based on SASA signifies how much the interface was occupied by a residue type as opposed to the surface. Cytoplasmic proteins clearly displayed a distinct profile where hydrophobic residues had greater tendencies for the interface than polar ones, which preferred the surface. For membrane proteins, hydrophobic residues preferred the interface support over the interior at the soluble side of the membrane. Polar residues displayed a slight inclination toward the interface in the lipid environment. Protrusion index profiles indicated that interface rim residues had the highest protrusion, as opposed to either surface or interface core residues. The distinction was especially noticeable for the cytoplasmic beta category. Membrane complexes displayed the lowest protrusion everywhere, and the distinction between the interface and surface diminished noticeably.

Surface roughness and planarity undoubtedly outperformed SASA and protrusion index for distinguishing the interface from the surface. For all residues in all categories, both roughness and planarity increased at the interface. These two descriptors clearly distinguished the interface, which accommodated a rugged surface that would favor several short-range vdW contacts and hydrogen bonds and a planar surface for shape complementarity. SASA became a strong discriminating feature between interface and surface for cytoplasmic beta complexes, where the mean value of the interface core was 84.2 Å^2^ lower than that of the surface.

The highest frequency of contacts was identified between hydrophobic residue pairs that involved Ile, Phe, Val, and Leu. Especially, one in every three contacts at the lipid part of the interface involved Leu. Leu was frequently observed in most contacts in all categories, yet its abundance in membrane complexes was noteworthy. Despite the abundance of hydrophobic contacts at the interface, the likelihood of observing a polar–polar contact was the strongest. This was mostly evident when the environment of the interface was lipid. On the other hand, no such strong preference existed among hydrophobic residues which associated randomly inside the lipid bilayer.

The structural descriptors highlighted in this work will provide a foundation for the development of an interface prediction algorithm, where different machine learning algorithms will be employed. Thus, for each category, the best-performing machine learning algorithm will be selected. A similar approach was supported by the study of Wang et al.,^32^ which emphasized the importance of employing different machine learning methods to predict different protein–protein interface patterns. However, their classification was based on functional differences such as enzyme–inhibitor, enzyme–substrate, enzyme–regulatory chain, G-protein-containing, etc. The novelty of our study lies in the categorization of our database based on the effect of secondary structure and environment, which are the two major factors affecting the interaction site of monomeric units.

## Data Availability

Database files and source codes for analysis used in this study are provided in the Supporting Information.

## Supporting Information Available

PDB IDs of the protein complexes grouped into three categories based on the secondary structure type of the interface and the surrounding environment (Supplementary Table 1); effect size for SASA distribution profiles between surface and rim regions (Supplementary Table 2); effect size for SASA distribution profiles between surface and core regions (Supplementary Table 3); propensity based on SASA values for different residue types in different categories (Supplementary Figure 1); distribution profile of SASA values of each residue type in surface, core and rim regions in different categories (Supplementary Figure 2); distribution profile of protrusion index values of each residue type in surface, core and rim regions in different categories (Supplementary Figure 3); surface roughness calculated for different patch radius in cytoplasmic alpha complexes (Supplementary Figure 4); surface planarity calculated for different patch radius in cytoplasmic alpha complexes (Supplementary Figure 5); distribution profile of roughness values of each residue type in surface, core and rim regions in different categories (Supplementary Figure 6); distribution profile of planarity values of each residue type in surface, core, and rim regions in different categories (Supplementary Figure 7); observed frequencies of contacts between residues from two monomers at the interface (Supplementary Figure 8) (PDF)
Source codes (ZIP)
Database files (TAR)

## Supplementary Material

ci4c01233_si_002.zip

ci4c01233_si_003.tar

## References

[ref1] Chothia C.; Janin J. Principles of protein-protein recognition. Nature 1975, 256, 705–708. 10.1038/256705a0. 1153006

[ref2] Dill K. A. Dominant forces in protein folding. Biochemistry 1990, 29, 7133–7155. 10.1021/bi00483a001. 2207096

[ref3] Young L.; Jernigan R. L.; Covell D. G. A role for surface hydrophobicity in protein-protein recognition. Protein Sci. 1994, 3, 717–729. 10.1002/pro.5560030501. 8061602 PMC2142720

[ref4] Jones S.; Thornton J. M. Protein-protein interactions: a review of protein dimer structures. Prog. Biophys. Mol. Biol. 1995, 63, 31–65. 10.1016/0079-6107(94)00008-W. 7746868

[ref5] Janin J.; Miller S.; Chothia C. Surface, subunit interfaces and interior of oligomeric proteins. J. Mol. Biol. 1988, 204, 155–164. 10.1016/0022-2836(88)90606-7. 3216390

[ref6] Argos P. An investigation of protein subunit and domain interfaces. Protein Eng. 1988, 2, 101–113. 10.1093/protein/2.2.101. 3244692

[ref7] Glaser F.; Steinberg D. M.; Vakser I. A.; Ben-Tal N. Residue Frequencies and Pairing Preferences at Protein-Protein Interfaces. Proteins: Struct., Funct., Genet. 2001, 43, 89–102. 10.1002/1097-0134(20010501)43:2<89::AID-PROT1021>3.0.CO;2-H. 11276079

[ref8] Bernstein F. C.; Koetzle T. F.; Williams G. J. B.; Meyer E. F.; Brice M. D.; Rodgers J. R.; Kennard O.; Shimanouchi T.; Tasumi M. The protein data bank: A computer-based archival file for macromolecular structures. J. Mol. Biol. 1977, 112, 535–542. 10.1016/S0022-2836(77)80200-3. 875032

[ref9] Lomize M. A.; Pogozheva I. D.; Joo H.; Mosberg H. I.; Lomize A. L. OPM database and PPM web server: resources for positioning of proteins in membranes. Nucleic Acids Res. 2012, 40 (D1), D370–D376. 10.1093/nar/gkr703. 21890895 PMC3245162

[ref10] Eswar N.; Webb B.; Marti-Renom M. A.; Madhusudhan M. S.; Eramian D.; Shen M. Y.; Pieper U.; Sali A. Comparative protein structure modeling using Modeller. Curr. Protoc. Bioinf. 2006, 15, 5.6.1–5.6.30. 10.1002/0471250953.bi0506s15. PMC4186674 18428767

[ref11] Martı-Renom M. A.; Stuart A. C.; Fiser A.; Sanchez R.; Melo F.; Sali A. Comparative protein structure modeling of genes and genomes. Annu. Rev. Biophys. Biomol. Struct. 2000, 29, 291–325. 10.1146/annurev.biophys.29.1.291. 10940251

[ref12] Madeira F.; Pearce M.; Tivey A. R. N.; Basutkar P.; Lee J.; Edbali O.; Madhusoodanan N.; Kolesnikov A.; Lopez R. Search and Sequence Analysis tools services from EMBL-EBI in 2022. Nucleic Acids Res. 2022, 50 (W1), W276–W279. 10.1093/nar/gkac240. 35412617 PMC9252731

[ref13] Humphrey W.; Dalke A.; Schulten K. VMD: Visual molecular dynamics. J. Mol. Graphics 1996, 14, 33–38. 10.1016/0263-7855(96)00018-5. 8744570

[ref14] Shrake A.; Rupley J. A. Environment and exposure to solvent of protein atoms. Lysozyme and insulin. J. Mol. Biol. 1973, 79, 351–371. 10.1016/0022-2836(73)90011-9. 4760134

[ref15] Levy E. D. A Simple Definition of Structural Regions in Proteins and Its Use in Analyzing Interface Evolution. J. Mol. Biol. 2010, 403, 660–670. 10.1016/j.jmb.2010.09.028. 20868694

[ref16] Miller S.; Janin J.; Lesk A. M.; Chothia C. Interior and surface of monomeric proteins. J. Mol. Biol. 1987, 196, 641–656. 10.1016/0022-2836(87)90038-6. 3681970

[ref17] Pintar A.; Carugo O.; Pongor S. Cx, an algorithm that identifies protruding atoms in proteins. Bioinformatics 2002, 18, 980–984. 10.1093/bioinformatics/18.7.980. 12117796

[ref18] Pontius J.; Richelle J.; Wodak S. J. Deviations from Standard Atomic Volumes as a Quality Measure for Protein Crystal Structures. J. Mol. Biol. 1996, 264, 121–136. 10.1006/jmbi.1996.0628. 8950272

[ref19] Jones S.; Thornton J. M. Analysis of protein-protein interaction sites using surface patches. J. Mol. Biol. 1997, 272, 121–132. 10.1006/jmbi.1997.1234. 9299342

[ref20] Lewis M.; Rees D. C. Fractal surfaces of proteins. Science 1985, 230, 1163–1165. 10.1126/science.4071040. 4071040

[ref21] Speer S. L.; Zheng W.; Jiang X.; Chu I-T.; Guseman A. J.; Liu M.; Pielak G. J.; Li C. The intracellular environment affects protein-protein interactions. Proc. Natl. Acad. Sci. U.S.A. 2021, 118 (11), e2019918118 10.1073/pnas.2019918118. 33836588 PMC7980425

[ref22] Eisenberg D.; Schwarz E.; Komaromy M.; Wall R. Analysis of Membrane and Surface Protein Sequences with the Hydrophobic Moment Plot. J. Mol. Biol. 1984, 179, 125–142. 10.1016/0022-2836(84)90309-7. 6502707

[ref23] Miller S.; Lesk A. M.; Janin J.; Chothia C. The accessible surface area and stability of oligomeric proteins. Nature 1987, 328, 834–836. 10.1038/328834a0. 3627230

[ref24] Bogan A. B.; Thorn K. S. Anatomy of hot spots in protein interfaces. J. Mol. Biol. 1998, 280, 1–9. 10.1006/jmbi.1998.1843. 9653027

[ref25] Chothia C. H. Structural invariants in protein folding. Nature 1975, 254, 304–308. 10.1038/254304a0. 1118010

[ref26] Kruschke J. K. Bayesian estimation supersedes the *t* test. J. Exp. Psychol. Gen. 2013, 142, 573–603. 10.1037/a0029146. 22774788

[ref27] Martins J. M.; Ramos R. M.; Pimenta A. C.; Moreira I. S. Solvent-accessible surface area: How well can be applied to hot-spot detection?. Proteins 2014, 82 (3), 479–490. 10.1002/prot.24413. 24105801

[ref28] Eisenberg D.; Weiss R. M.; Terwilliger T. C.; Wilcox W. Hydrophobic moments and protein structure. Faraday Symp. Chem. Soc. 1982, 17, 109–120. 10.1039/fs9821700109.

[ref29] Pettit F. K.; Bowie J. U. Protein surface roughness and small molecular binding sites. J. Mol. Biol. 1999, 285, 1377–1382. 10.1006/jmbi.1998.2411. 9917382

[ref30] Wu F.; Towfic F.; Dobbs D.; Honavar V. Analysis of protein protein dimeric interfaces. In Proceedings of the 2007 IEEE International Conference on Bioinformatics and Biomedicine (BIBM 2007); IEEE, 2007.

[ref31] Yan C.; Wu F.; Jernigan R. L.; Dobbs D.; Honavar V. Characterization of protein-protein interfaces. Protein J. 2008, 27 (1), 59–70. 10.1007/s10930-007-9108-x. 17851740 PMC2566606

[ref32] Wang W.; Yang Y.; Yin J.; Gong X. Different protein-protein interface patterns predicted by different machine learning method. Sci. Rep. 2017, 7, 16023 10.1038/s41598-017-16397-z. 29167570 PMC5700192

