# Supplementary material for: Structural Descriptors for Subunit Interface Regions in Homodimers: Effect of Lipid Membrane and Secondary Structure Type
Source: J Chem Inf Model. 2025 Mar 27;65(7):3117–26. doi: 10.1021/acs.jcim.4c01233 (PMC12004529; doi:10.1021/acs.jcim.4c01233)
Supplement: Supplementary file 1 — ci4c01233_si_001.pdf [file ci4c01233_si_001.pdf]

SUPPORTING INFORMATION

Structural Descriptors for Subunit Interface Regions in Homodimers: Effect of Lipid Membrane and Secondary Structure Type

Aslı Yüksek[1], Batuhan Yıkınç[1], İrem Nayır[1], Defne Alnıgeniş[1], Vahap Gazi Fidan[1], Tayyip Topuz[2], Ebru Demet Akten[1*]

[1]Department of Molecular Biology and Genetics, Faculty of Engineering and Natural Sciences, Kadir Has University, 34083, Fatih, Istanbul, Turkey

[2]Graduate School of Studies, Ph.D. program of Computer Engineering, Kadir Has University, 34083, Fatih, Istanbul, Turkey

[*]Corresponding Author

demet.akten@khas.edu.tr

+90 212 533 65 32 (x1350)

https://orcid.org/0000-0002-0358-3171

Supplementary Table 1. PDB id's of the protein complexes grouped into three categories based on the secondary structure type of the interface and the surrounding environment.

Membrane (478)	1a25-1aa7-1aqt-1aua-1avv-1btn-1c3i-1cdt-1dfn-1djt-1dqt-1dxr-1ea5-1eys-1fhx-1g5z-1hfa-1hlg-1hyn-1kf6-1kpl-1ktj-1kxi-1l7v-1l9b-1lbq-1lpp-1m7r-1mhs-1mm9-1oja-1ots-1pfo-1pp2-1py9-1q16-1qs8-1rk4-1t6m-1tjj-1tu5-1umv-1uzg-1v3e-1vf6-1vsg-1wdz-1xu7-1xx1-1y0g-1y5m-1z9h-1zoy-1zwx-2a65-2bdm-2bhv-2bs2-2c08-2cfu-2d4c-2dtc-2efk-2f1m-2fic-2h4c-2h88-2hih-2hyd-2mpn-2nq2-2onk-2p0m-2q63-2qk7-2qpt-2r83-2rj6-2v0o-2vpx-2vsg-2wli-2x3v-2x72-2xq2-2yvx-2z0v-2z73-2zup-3ajm-3ayg-3b5x-3b60-3bl8-3brx-3c0m-3d31-3d34-3dhw-3dhx-3dih-3ebw-3fo5-3fqm-3fvq-3g8g-3gro-3h90-3hl4-3hpe-3i03-3icv-3j08-3j1z-3k8i-3kgc-3l1l-3lll-3m31-3nd0-3ne5-3nvo-3odj-3oe0-3org-3p0c-3p94-3pjz-3puw-3q34-3qe6-3qni-3qnq-3rbx-3rfi-3sx6-3t0r-3ug9-3ukm-3utv-3vne-3w54-3wme-3x3b-3zdq-3zux-4a01-4ab0-4av3-4avm-4bdt-4bw5-4bwz-4cz8-4czb-4d9o-4djh-4djk-4dkl-4fkb-4fmm-4g1u-4g33-4gpo-4h8s-4huq-4hzu-4io2-4j72-4j7c-4jch-4jkv-4kbr-4klr-4mnd-4mrs-4ms3-4myc-4nc9-4njn-4nsw-4o6m-4o6y-4o9s-4oh3-4on3-4opm-4ors-4p2p-4p42-4pl0-4q2e-4qid-4qin-4qn9-4qnc-4qnd-4r0c-4r1i-4rfs-4ri2-4rng-4rp8-4rue-4ry2-4ryi-4tl3-4tqu-4u14-4u9n-4uc1-4v2o-4wis-4x5m-4ymu-4ysx-4zhk-5a1s-5a3v-5a40-5a43-5a50-5b57-5bov-5c73-5c76-5cmk-5d3m-5d92-5dhg-5do7-5doq-5dyy-5f19-5fn3-5fqu-5fwx-5gko-5h6b-5hh0-5i6c-5i7k-5iji-5iws-5ixg-5jnq-5khn-5kn7-5l22-5lil-5ly9-5mkk-5mrw-5o5e-5o9h-5oc9-5och-5oge-5on7-5oyb-5tfv-5tkg-5tqq-5u1d-5uhq-5unf-5uvg-5v6p-5wd6-5x3x-5x5y-5x61-5xls-5xmj-5xqp-5xu1-5y78-5yjy-5yqq-5z1f-5zih-5zlg-5zov-6a2w-6agi-6b3i-6b87-6bgi-6bq0-6btm-6bw5-6bym-6c5w-6c9a-6cb2-6csm-6d0j-6dz7-6e1m-6e7r-6eid-6f0k-6fnp-6fv7-6h5a-6i1z-6ig4-6ijz-6iql-6is6-6iu3-6jbh-6jbj-6kob-6koi-6ksi-6kuw-6l3h-6l47-6l85-6lod-6m1y-6m23-6m32-6m49-6m96-6mfo-6mhu-6n1g-6n51-6n52-6nf4-6nf6-6npl-6nq0-6nt5-6nt6-6nwd-6o84-6oce-6oht-6oop-6p2j-6p6i-6p6j-6pnw-6pt3-6pv7-6q42-6qp6-6qq5-6qti-6qv0-6qvb-6r72-6rko-6rtc-6rv3-6s3k-6su3-6tej-6tek-6tqe-6uo8-6usu-6uxm-6vja-6vp0-6vqt-6vyn-6w2y-6w7b-6w8n-6wk5-6wm5-6wtw-6wu3-6wu4-6x3v-6xdc-6xfj-6xjh-6xjo-6xyu-6y3c-6y9a-6ykr-6yuz-6z0f-6z3y-6z79-6zg3-7ad3-7ahc-7ahd-7aip-7arh-7ark-7b1k-7b4l-7b9f-7bp3-7bve-7bvf-7bxu-7cad-7cag-7cff-7ch1-7ch6-7chf-7cj3-7d10-7d5i-7d7q-7d7r-7d99-7dgd-7drj-7dsv-7e6t-7e9g-7e9h-7ehl-7epa-7epc-7epd-7ezc-7f3t-7f4f-7f73-7fh1-7j7t-7jk8-7jsj-7kyo-7lb8-7lgu-7m1u-7m33-7m94-7mbz-7n58-7nkz-7occ-7ojh-7oz1-7p1k-7p34-7p54-7p5c-7p5j-7psd-7psl-7qeo-7qp9-7rit-7rtm-7she-7stl-7tak-7tm9-7vfi-7vgf-7voj-7vr1-7vwc-7w6k-7wiv-7wjm-7wks-7y5g-7z6m-7zgo-7znu-8b4o-8dku-8f4n
Cytoplasmic alpha-helical (422)	11ba-139l-17gs-1a17-1a43-1ae7-1afr-1aj8-1al6-1aok-1aw1-1b43-1b57-1b8g-1b9b-1bmt-1btm-1bxk-1chu-1cqx-1csm-1d2r-1ddz-1dli-1dvj-1e2h-1e7d-1ee1-1ee8-1ek6-1f05-1f0y-1fp3-1g1a-1g6w-1gmz-1gqi-1gsz-1gu6-1gy8-1h1y-1hn4-1hti-1i2n-1i45-1i4s-1idt-1iom-1is2-1iyh-1j1a-1j1j-1j93-1j9i-1jfa-1jk0-1jqo-1jzt-1ka8-1kbl-1kcf-1kea-1kgn-1kv5-1lns-1lon-1lxy-1m6j-1mo0-1n0h-1n1b-1n1q-1n3l-1nht-1nox-1np3-1nvd-1o0w-1o17-1o5h-1o5x-1oc2-1ogk-1orr-1oyf-1p5z-1p7n-1p9b-1pvd-1pyd-1ql0-1qmg-1r2f-1r3q-1r8g-1req-1rqg-1rqi-1rvg-1rxq-1s9c-1sb8-1so2-1szq-1tj7-1txg-1tya-1tz9-1ubv-1uda-1udu-1ukw-1ulh-1uzr-1v4e-1v4v-1vbm-1vgv-1via-1vip-1vjt-1w07-1w2y-1w53-1ww1-1x0x-1x94-1xg7-1xng-1xqi-1xzw-1y42-1y44-1yhk-1yi8-1ynf-1yv5-1yxy-1yyq-1zch-1zkw-1zl7-2a6b-2a84-2a9u-2ag6-2ahr-2ao2-2aq0-2b0j-2b5d-2b67-2bwj-2c20-2c5e-2cya-2dh4-2e1e-2e21-2eja-2el7-2ev1-2f1k-2flo-2fon-2g36-2g4l-2g5c-2gf2-2ggl-2ggp-2gi3-2gn0-2gr9-2h9c-2hbv-2hrc-2hun-2hvg-2i22-2inf-2isv-2iz0-2j07-2jas-2k1r-2o70-2ocp-2oej-2oma-2opi-2p5u-2par-2pbp-2pid-2pla-2pv3-2pv7-2pw3-2pzb-2qaf-2rd3-2v6k-2w8z-2xiq-2xpk-2yaz-2yy2-2yy5-2yyn-2zvi-2zya-3a04-3b9r-3bem-3beo-3bnx-3bsm-3cky-3cqh-3ct2-3d4j-3der-3dg7-3dpi-3dzc-3e7d-3enk-3f0n-3fef-3fiu-3fk4-3fq7-3g7i-3gwl-3hfr-3hmq-3hv0-3hwk-3i05-3i4k-3i6e-3itm-3jxe-3kbp-3kdy-3kt0-3lom-3m31-3m5w-3mzn-3n2s-3nav-3npk-3nz4-3o3m-3odm-3olj-3prh-3q58-3qn3-3qtp-3r11-3r12-3r89-3rk8-3ro6-3ru7-3ssa-3sz3-3tw9-3tze-3uu0-3vgj-3w6z-3wdk-3wjk-3zcd-4aql-4au1-4bof-4cvp-4d7a-4d8f-4e0h-4f1w-4f66-4fkz-4g8t-4i71-4j5p-4j75-4jwt-4kqx-4l5l-4lkq-4lzj-4mf5-4mpf-4n5f-4neq-4ny7-4omg-4oun-4ppu-4q16-4q9o-4qss-4ri6-4u04-4u83-4wsh-4x4w-4yh2-4yio-4ywq-4yym-5ag2-5ayp-5enz-5f05-5f23-5ihx-5je8-5jef-5k3g-5k3j-5ks7-5lcz-5ltf-5lvr-5mux-5o5t-5syu-5ts9-5uq9-5uqo-5v0i-5vkw-5w6y-5whr-5wp0-5y8g-5y9z-5zka-6a71-6at7-6bej-6byq-6c5c-6cnz-6dfu-6ebo-6egk-6ep7-6ex3-6ex4-6ezu-6fqx-6gsb-6gza-6i2z-6iv7-6mtk-6ncr-6pro-6qo5-6r6u-6riv-6vo2-6vso-6yka-6zjk-7axy-7e1l-7el8-7eqe-7eqf-7exe-7fg6-7kvh-7lbj-7m32-7my0-7my6-7n9o-7ngj-7o04-7oom-7owv-7pp7-7pt0-7pv1-7q16-7qaa-7qbk-7qoc-7qp9-7r62-7r6u-7s3z-7s76-7sfo-7tdd-7te7-7twa-7vg4-7vwe-7vwt-7wbr-7wxz-7wze-7y0y-7yxh-7zrn-8czj-8dsy-8du6-8dub-8eq8-8gxl-8nse
Cytoplasmic beta-strand (411)	12e8-1a6u-1a78-1a7n-1aaq-1ac6-1at0-1b8m-1bdy-1bet-1bso-1c12-1c1f-1c5b-1cd8-1cdt-1cfv-1cqk-1d2s-1d5i-1d9k-1dbn-1dl7-1dn0-1dqd-1dql-1dqt-1eap-1eeq-1eym-1f4w-1f86-1f9k-1fh5-1fl3-1ggb-1gnz-1h5b-1h91-1h9w-1hq4-1hza-1i3g-1iam-1igi-1igm-1j05-1jfq-1jhe-1jpy-1jv5-1kcu-1l4i-1mfa-1mi8-1mkk-1moe-1mqk-1my5-1n7m-1nj9-1njh-1nzi-1o4t-1oaq-1opa-1ouw-1p4i-1pdg-1pfs-1py9-1q0x-1q8m-1qc6-1qjg-1qmj-1qu0-1rur-1rzi-1sda-1se8-1spp-1tvd-1tza-1uis-1usc-1usr-1uww-1uxm-1uzy-1vh4-1vhz-1vj2-1vsc-1w6m-1wlc-1wlg-1wlh-1wq9-1wz1-1xe6-1xhn-1xko-1xrk-1xsq-1xvs-1y0g-1yai-1ylx-1yt5-1yzz-1z24-1z52-1zhq-1zm1-1zmi-1zps-1zso-2amu-2aq6-2ar6-2axh-2b4h-2bdr-2c11-2c7w-2cg6-2ck2-2co3-2cxk-2d6k-2d6l-2dct-2dud-2e5y-2f4p-2fhq-2fvu-2fyr-2gi7-2gvb-2gx9-2h1t-2h2n-2hl0-2hq7-2huh-2i02-2i2l-2i45-2i9x-2iep-2ig6-2ihp-2imm-2imz-2in5-2jjw-2ns9-2nu5-2o3o-2ooj-2ook-2oov-2ox5-2oyz-2ozj-2p1r-2pyt-2q03-2q3a-2r4i-2rde-2rh7-2snw-2v6h-2vkw-2vnv-2vzx-2wo0-2xr4-2y0o-2yd6-2yn3-2z77-2zdo-2zf3-2zgm-2zhp-2zmk-2zo6-35c8-3a6q-3axa-3b5g-3b76-3b83-3b9i-3bx9-3c2s-3c8x-3caf-3cdc-3cfx-3cnk-3cvi-3czz-3d82-3d9r-3dh5-3dui-3ec6-3ejz-3emm-3eo0-3eo6-3er7-3ew1-3exu-3f1n-3fc0-3fgy-3g5x-3gb3-3h2d-3hds-3hpe-3hqx-3hzp-3i2z-3i9i-3ia8-3ipf-3ipt-3iy4-3js1-3jx8-3k8a-3khf-3knb-3knf-3kyg-3l1o-3lgj-3lgm-3lsd-3lva-3lwc-3m3c-3m8c-3mce-3mit-3mqi-3nn8-3o0l-3osv-3pgz-3ps4-3q5t-3qs2-3rfh-3rkc-3rnk-3sed-3snm-3soy-3szh-3u0i-3u1s-3wea-3wud-3zhk-43c9-4a6s-4aj0-4apv-4auy-4axo-4azo-4b53-4b96-4be5-4c83-4d4t-4dz3-4g9m-4he4-4hpk-4jvw-4ko7-4lsd-4m6a-4mir-4n04-4npr-4pib-4q14-4q26-4q97-4q9c-4r4u-4rr6-4x4z-4xin-4z1b-4z27-4z6j-4zno-5a3l-5a9d-5aze-5b21-5b4m-5bnc-5cxo-5dd1-5dg2-5ews-5gal-5grv-5gvy-5h23-5h9q-5i8e-5jdj-5jny-5mh1-5moj-5mu8-5mvg-5mwj-5niv-5nld-5noi-5szf-5t4z-5t50-5tfs-5u38-5uel-5v00-5v52-5v77-5vga-5vvf-5w05-5w12-5wec-5wqv-5wsd-5x5x-5xg7-5xgt-5xhg-5xli-5xna-5xqw-5xrg-5y03-5ykv-5yl0-6a76-6amj-6aq6-6avn-6b3c-6b6i-6bpb-6c0m-6cqo-6d38-6d9g-6dwi-6dzn-6e20-6ebx-6f83-6gc2-6h3h-6hds-6hgu-6i1o-6j9o-6kjl-6l8t-6l98-6mee-6mg4-6mkb-6n3r-6niw-6p4y-6p79-6x7h-6y0w-6ya2-6z4u-7ah1-7byu-7c28-7c8p-7ezz-7jo7-7jxd-7kkz-7obf-7skn-7v4s-7vzo-7xmw-8dyg

Supplementary Table 2. Effect size (difference in means scaled by the pool estimates of standard deviation) for SASA distribution profiles between surface and rim regions.

	Residue Type	Cytoplasmic Alpha	Cytoplasmic Beta	Membrane LIPID	Membrane OUTSIDE
POLAR	ARG (-2.53)[&]	0.78 (28.56)	-0.42 (-13.65)	0.45 (18.12)	0.53 (20.22)
	LYS (-1.50)	0.48 (16.27)	-0.30 (-9.83)	0.25 (8.04)	0.42 (14.06)
	ASP (-0.90)	0.49 (12.99)	-0.16 (-4.69)	0.71 (14.76)	0.40 (10.78)
	GLN (-0.85)	0.55 (15.78)	-0.22 (-6.32)	0.71 (19.31)	0.41 (12.06)
	ASN (-0.78)	0.47 (13.06)	-0.57 (-15.19)	**0.95 (18.46)***	0.37 (10.24)
	GLU (-0.74)	0.47 (13.65)	-0.37 (-10.95)	**0.96 (25.20)***	0.42 (12.73)
	HIS (-0.40)	0.59 (18.10)	-0.41 (2.27)	0.64 (16.44)	0.40 (13.20)
	SER (-0.18)	0.51 (10.15)	-0.09 (-2.13)	0.63 (8.10)	0.48 (10.21)
	THR (-0.05)	0.53 (11.87)	-0.41 (-7.34)	0.62 (9.25)	0.51 (12.06)
HYDROPHOBIC	PRO (0.12)	0.54 (13.09)	-0.36 (-8.20)	0.65 (12.07)	0.48 (11.91)
	TYR (0.26)	**1.31 (38.25)***	-0.4 (-10.02)	**0.93 (29.28)***	0.75 (27.74)
	CYS (0.29)	**0.82 (22.13)***	**0.91 (23.30)***	**0.81 (12.88)***	0.49 (12.37)
	GLY (0.48)	0.42 (6.46)	-0.19 (-3.33)	0.68 (5.54)	0.60 (9.51)
	ALA (0.62)	0.59 (10.76)	-0.30 (-5.93)	0.74 (8.61)	0.42 (8.18)
	MET (0.64)	0.60 (21.50)	-0.35 (-13.13)	**0.82 (18.82)***	0.54 (20.41)
	TRP (0.81)	**1.03 (37.26)***	-0.26 (-8.50)	0.54 (19.32)	0.66 (29.88)
	LEU (1.06)	**1.22 (30.16)***	-0.29 (-6.91)	0.68 (14.87)	0.56 (16.74)
	VAL (1.08)	**0.88 (20.36)***	-0.46 (-8.21)	0.64 (10.63)	0.52 (12.83)
	PHE (1.19)	**1.05 (34.63)***	-0.14 (-4.33)	0.74 (20.09)	0.55 (20.01)
	ILE (1.38)	**0.95 (25.59)***	-0.44 (-8.84)	0.69 (13.65)	0.66 (18.64)

* effect size with a magnitude greater than 0.8 typed in bold.
[&] residue's normalized hydrophocity constants (Eisenberg *et al.* 1982).

Supplementary Table 3. Effect size (difference in means scaled by the pool estimates of standard deviation) for SASA distribution profiles between surface and core regions.

	Residue Type	Cytoplasmic Alpha	Cytoplasmic Beta	Hydrophobic TM	Soluble TM
POLAR	ARG (-2.53)[&]	-0.19 (-6.14)	**-2.79 (-79.72)**	-0.17 (-6.89)	-0.32 (-12.20)
	LYS (-1.50)	-0.39 (-12.59)	**-2.87 (-74.57)**	-0.43 (-12.99)	-0.39 (-13.22)
	ASP (-0.90)	-0.50 (-11.68)	**-2.79 (-63.35)**	0.08 (-1.61)	-0.35 (-8.89)
	GLN (-0.85)	-0.42 (-11.23)	**-2.98 (-72.74)**	-0.28 (-7.35)	-0.31 (-8.66)
	ASN (-0.78)	-0.39 (-10.23)	**-2.89 (-67.24)**	0.03 (0.58)	-0.38 (-9.69)
	GLU (-0.74)	-0.51 (-14.15)	**-2.99 (-74.08)**	-0.14 (-3.43)	-0.35 (-10.13)
	HIS (-0.40)	-0.37 (-10.98)	**-2.71 (-80.12)**	0.17 (5.03)	-0.38 (-11.99)
	SER (-0.18)	-0.42 (-7.55)	**-2.92 (-53.71)**	0.02 (0.27)	-0.37 (-7.03)
	THR (-0.05)	-0.20 (-4.24)	**-2.93 (-50.57)**	0.14 (2.16)	-0.29 (-6.08)
HYDROPHOBIC	PRO (0.12)	-0.19 (-4.59)	**-3.05 (-59.11)**	0.09 (1.69)	-0.25 (-6.31)
	TYR (0.26)	0.32 (9.86)	**-2.91 (-77.09)**	-0.08 (-2.39)	-0.01 (-0.53)
	CYS (0.29)	0.05 (1.25)	**-2.98 (-47.80)**	-0.29 (-4.05)	-0.26 (-5.83)
	GLY (0.48)	-0.45 (-6.53)	**-2.88 (-39.33)**	-0.03 (-0.21)	-0.40 (-5.86)
	ALA (0.62)	-0.40 (-6.51)	**-3.01 (-49.37)**	0.04 (0.49)	-0.41 (-7.22)
	MET (0.64)	-0.20 (-6.53)	**-2.72 (-91.45)**	0.14 (3.38)	-0.44 (-14.41)
	TRP (0.81)	0.17 (6.07)	**-3.10 (-90.34)**	-0.46 (-16.29)	-0.25 (-10.68)
	LEU (1.06)	0.16 (3.96)	**-3.08 (-68.93)**	-0.10 (-2.21)	-0.16 (-4.51)
	VAL (1.08)	0.01 (0.30)	**-3.11 (-54.06)**	-0.11 (-1.72)	-0.18 (-3.95)
	PHE (1.19)	0.20 (6.26)	**-2.93 (-82.96)**	-0.15 (-4.32)	-0.23 (-7.92)
	ILE (1.38)	0.05 (1.28)	**-2.99 (-62.81)**	0.02 (0.42)	-0.09 (-2.44)

* effect size with a magnitude greater than 0.8 typed in bold.
[&] residue's normalized hydrophocity constants (Eisenberg *et al.* 1982).

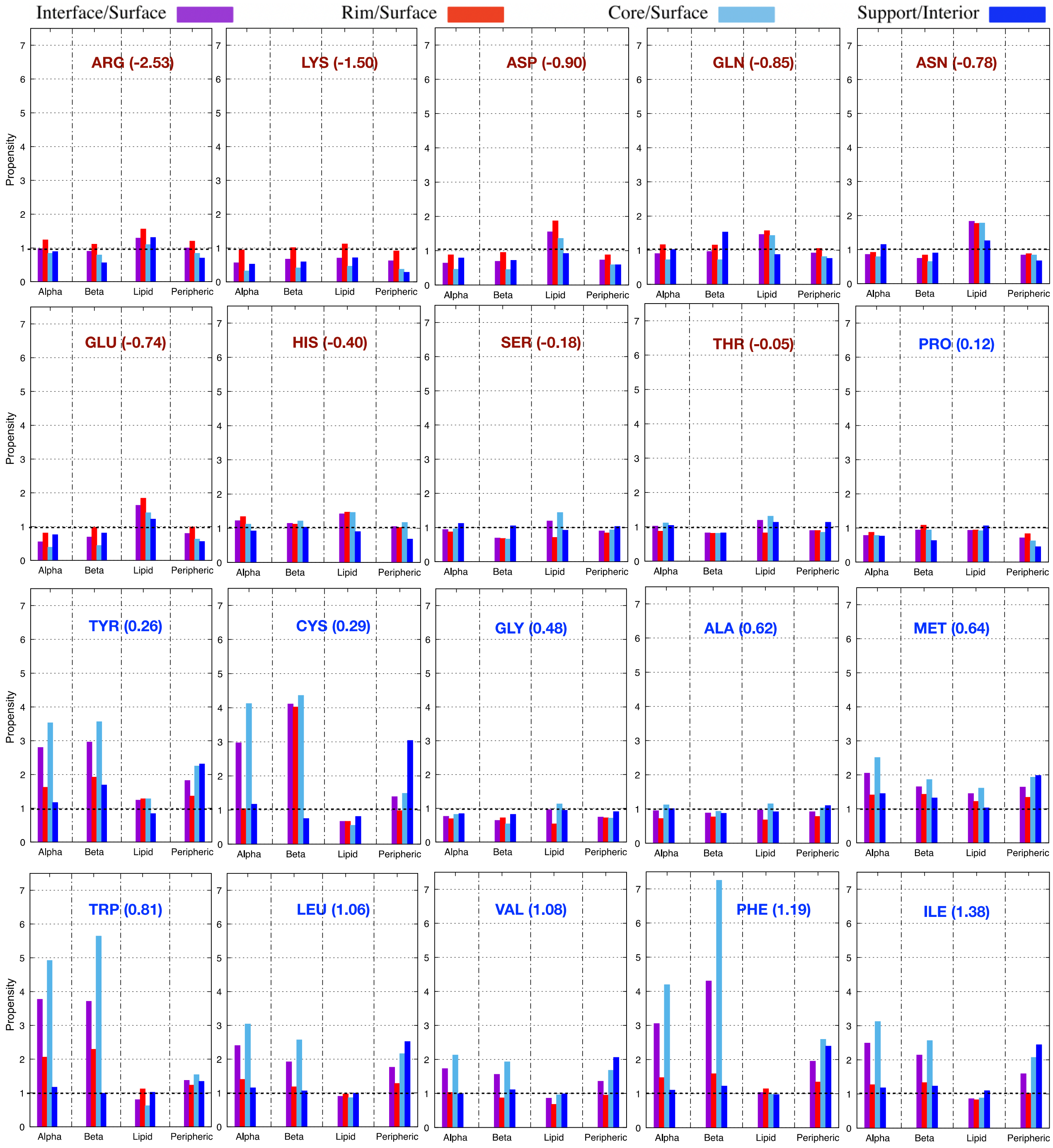

Supplementary Figure 1. Propensity based on SASA values for different residue types in different categories. See Methods section for details. Polar and hydrophobic residues highlighted with red and blue labels, respectively.

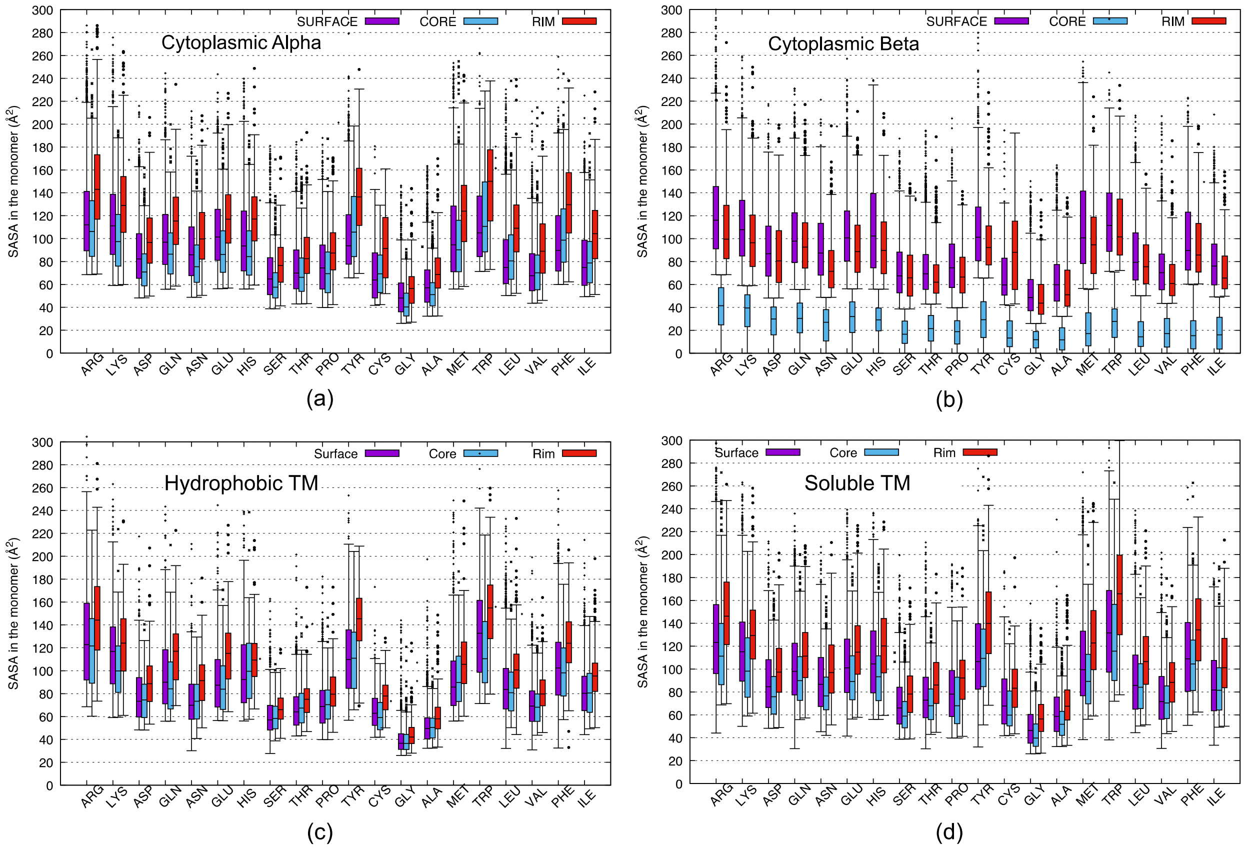

Supplementary Figure 2. Distribution profile of SASA values of each residue type in surface, core and rim regions in different categories.

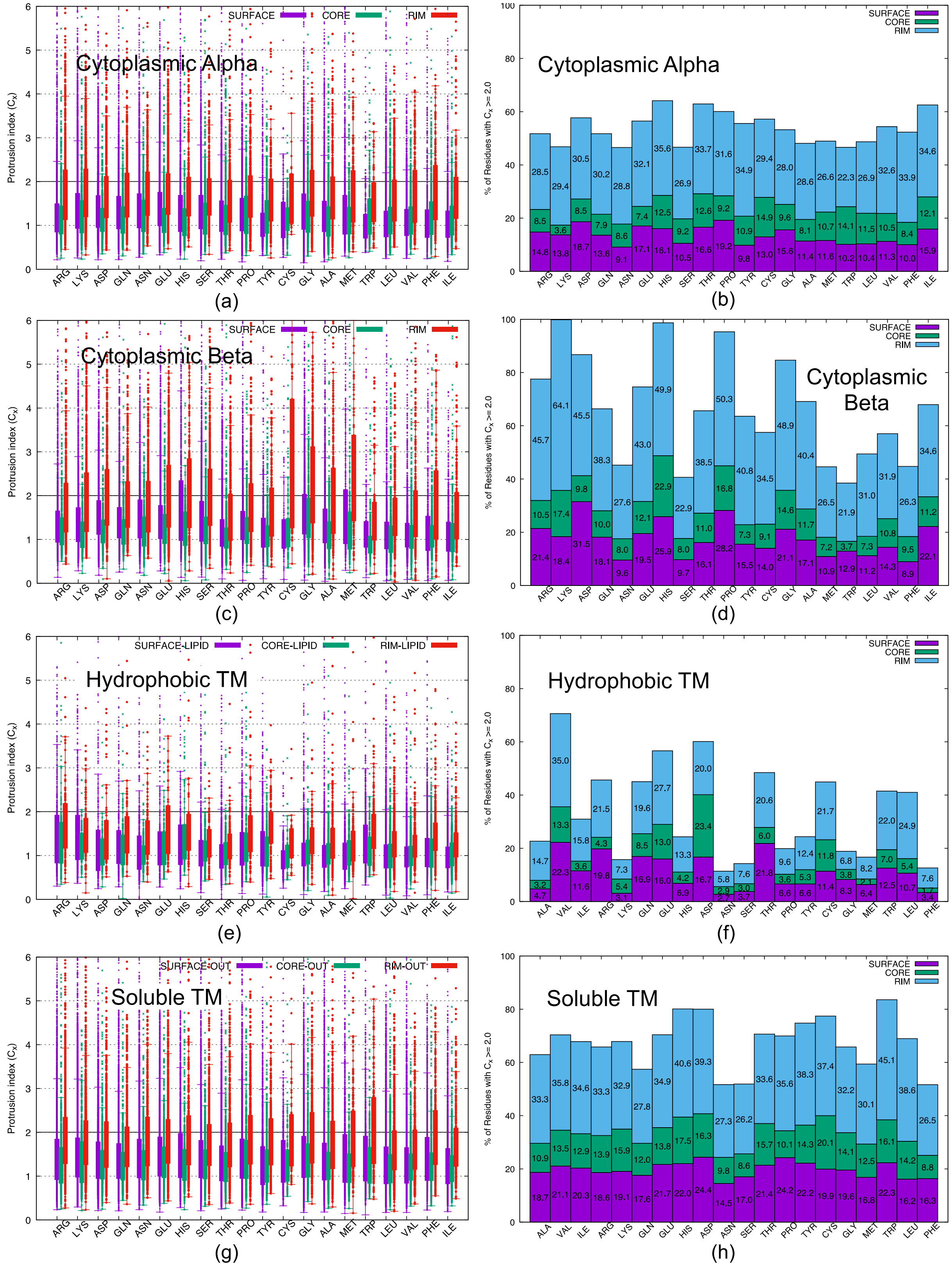

Supplementary Figure 3. (a),(c),(e),(g) Distribution profiles of protrusion index values of each residue type in surface, core and rim regions in different categories, (b),(d),(f),(h) Percent of residues with protrusion index values (C_x) greater than 2.0.

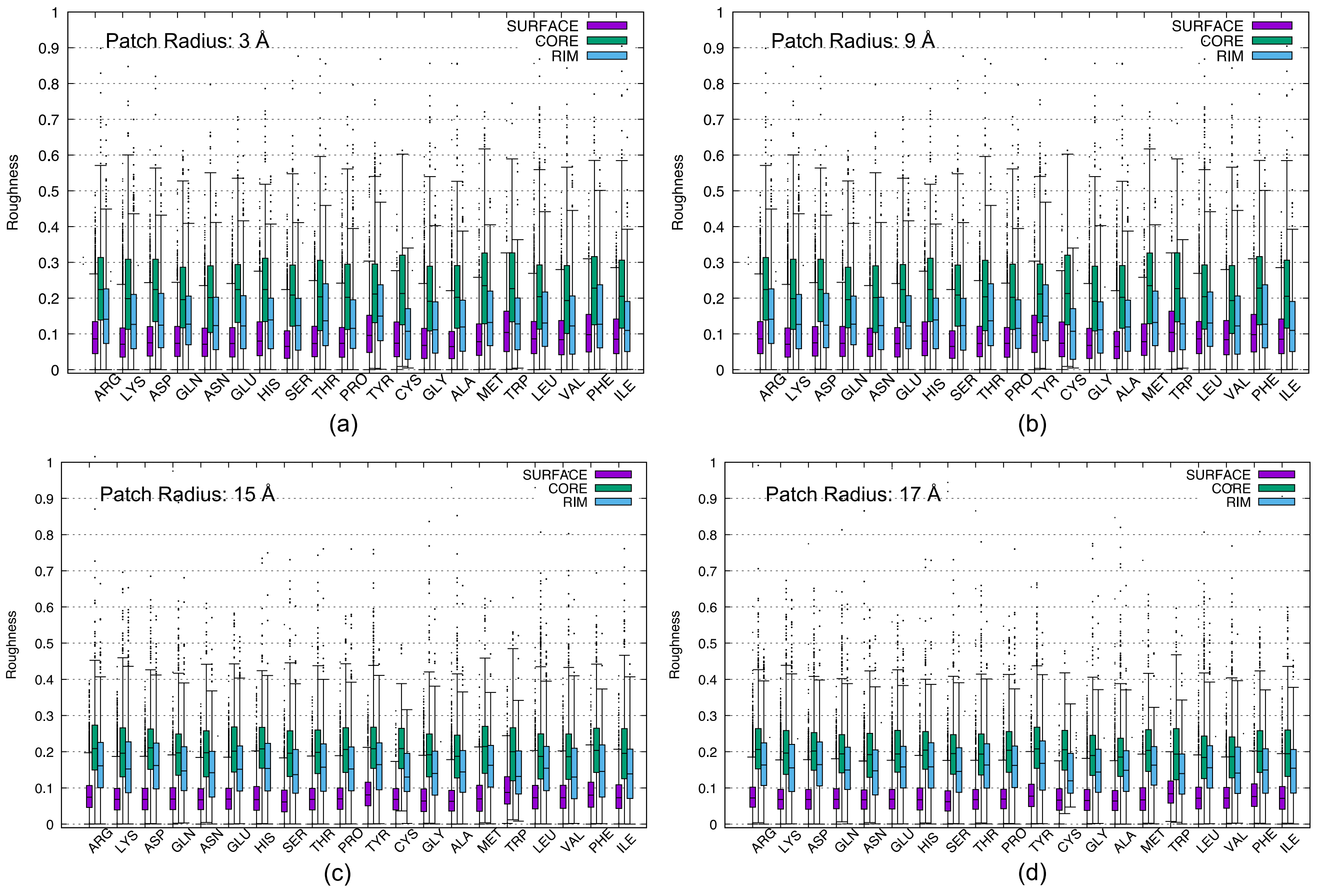

Supplementary Figure 4. Surface roughness calculated for different patch radius in cytoplasmic alpha complexes

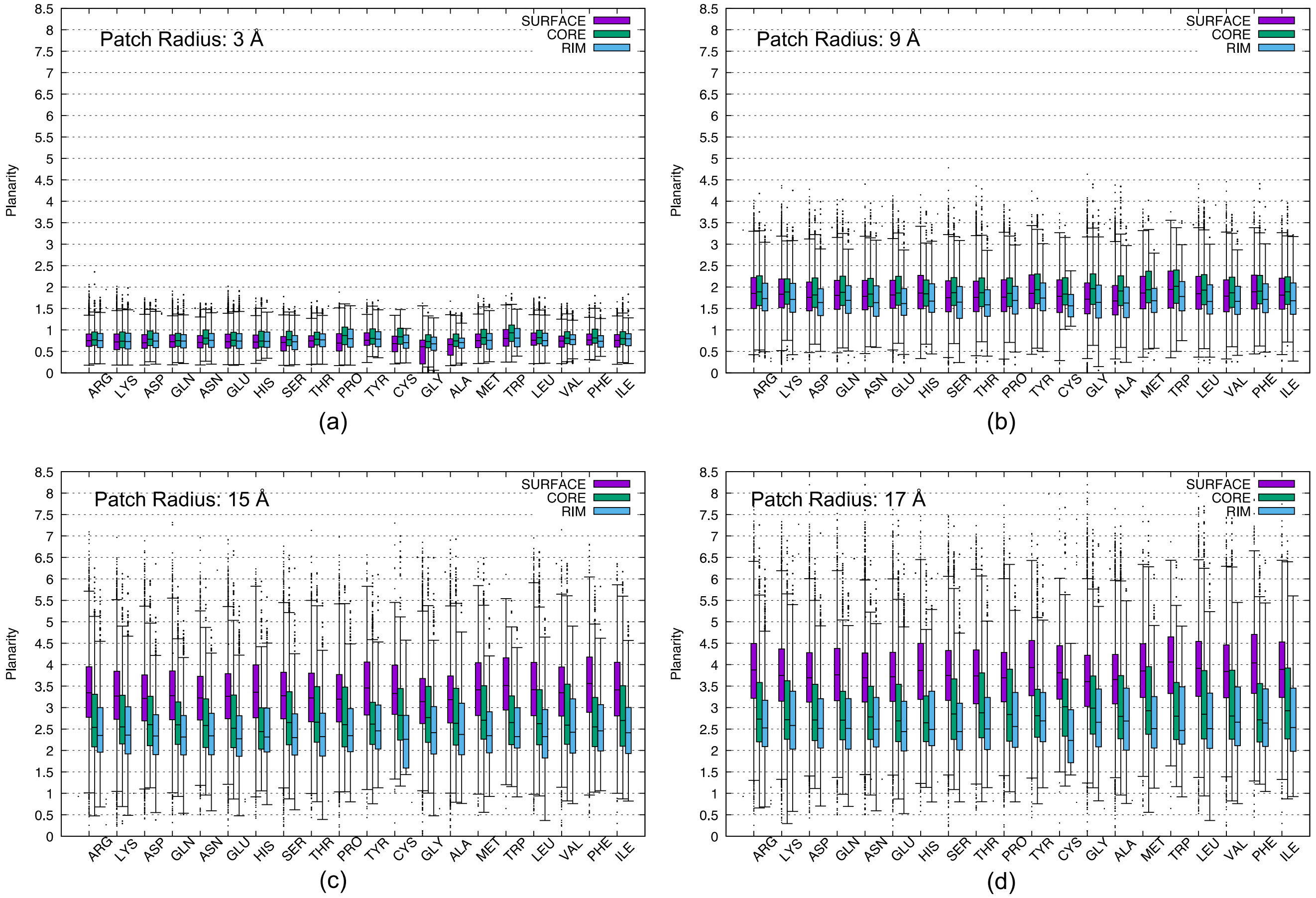

Supplementary Figure 5. Surface planarity calculated for different patch radius in cytoplasmic alpha complexes

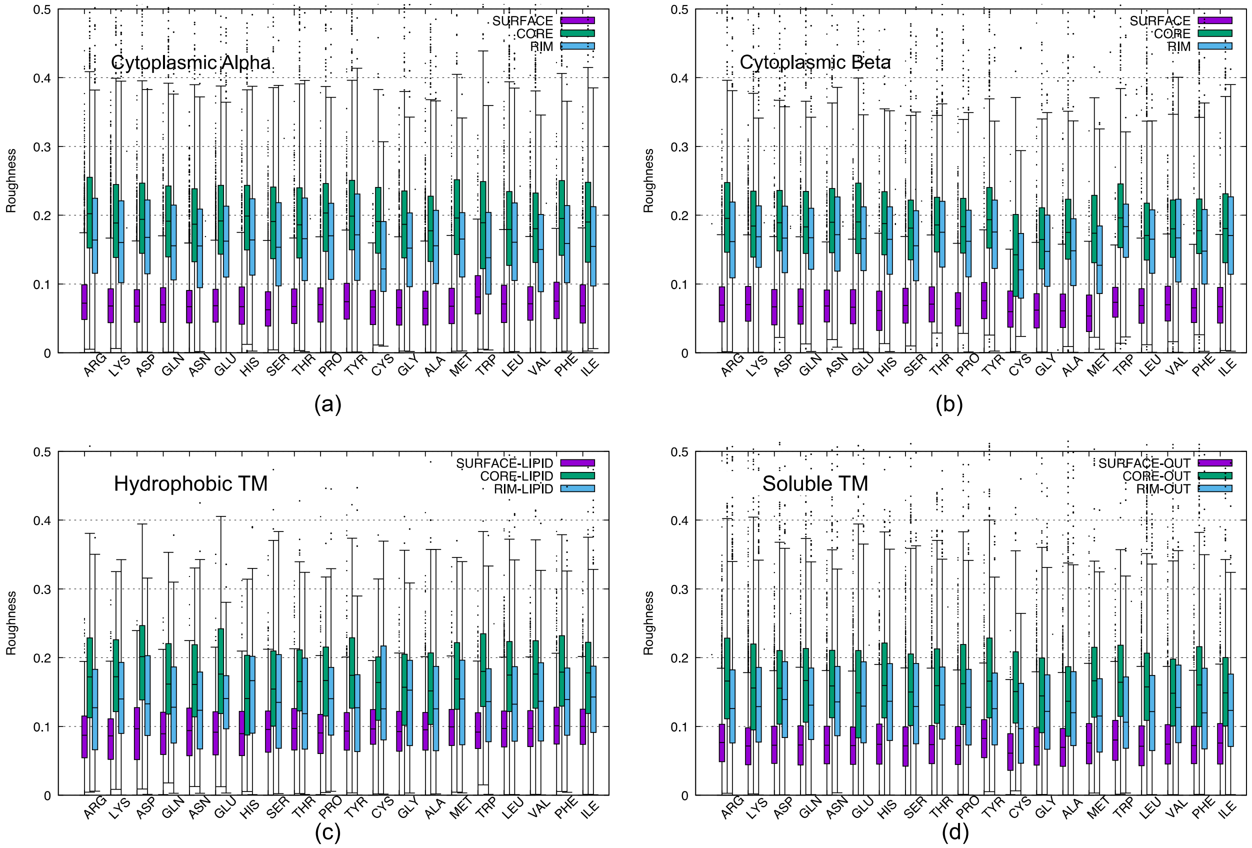

Supplementary Figure 6. Distribution profile of roughness values of each residue type in surface, core and rim regions in different categories

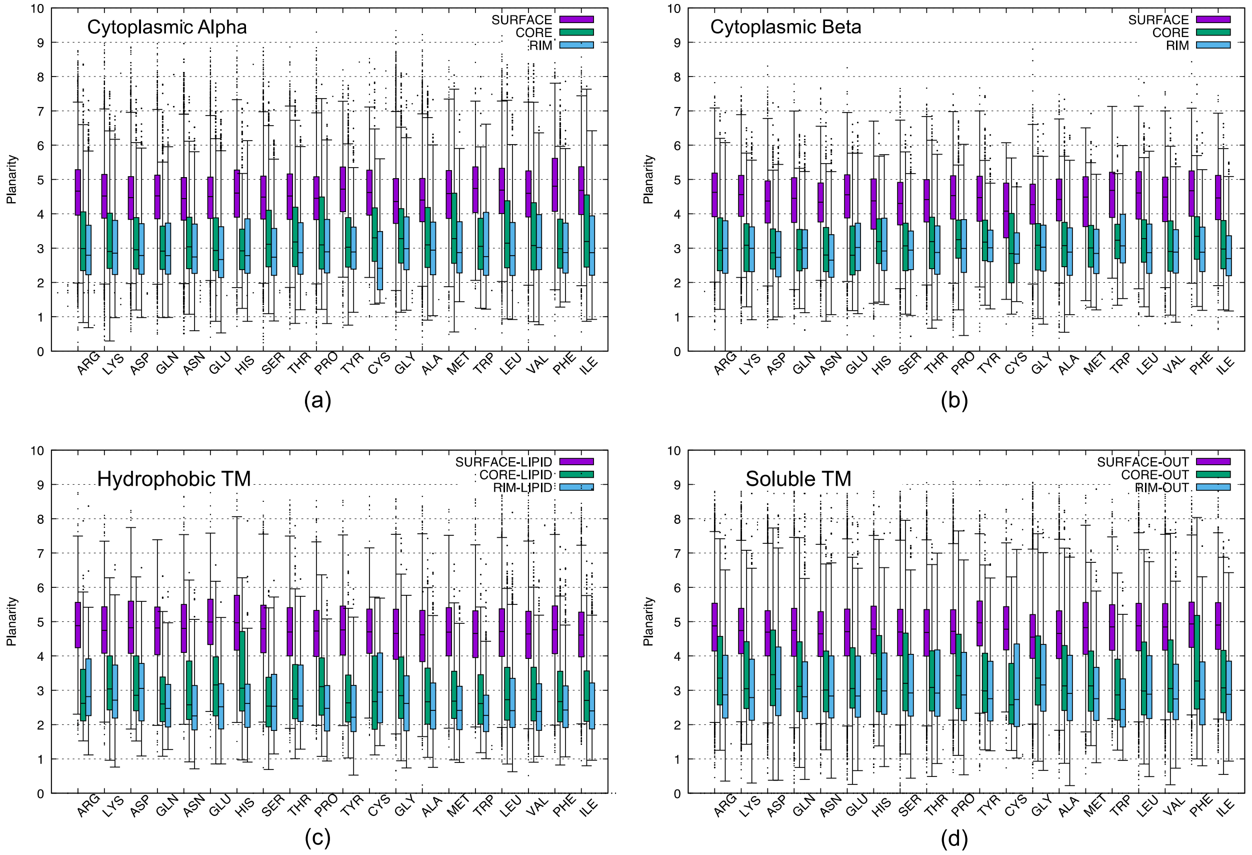

Supplementary Figure 7. Distribution profile of planarity values of each residue type in surface, core and rim regions in different categories

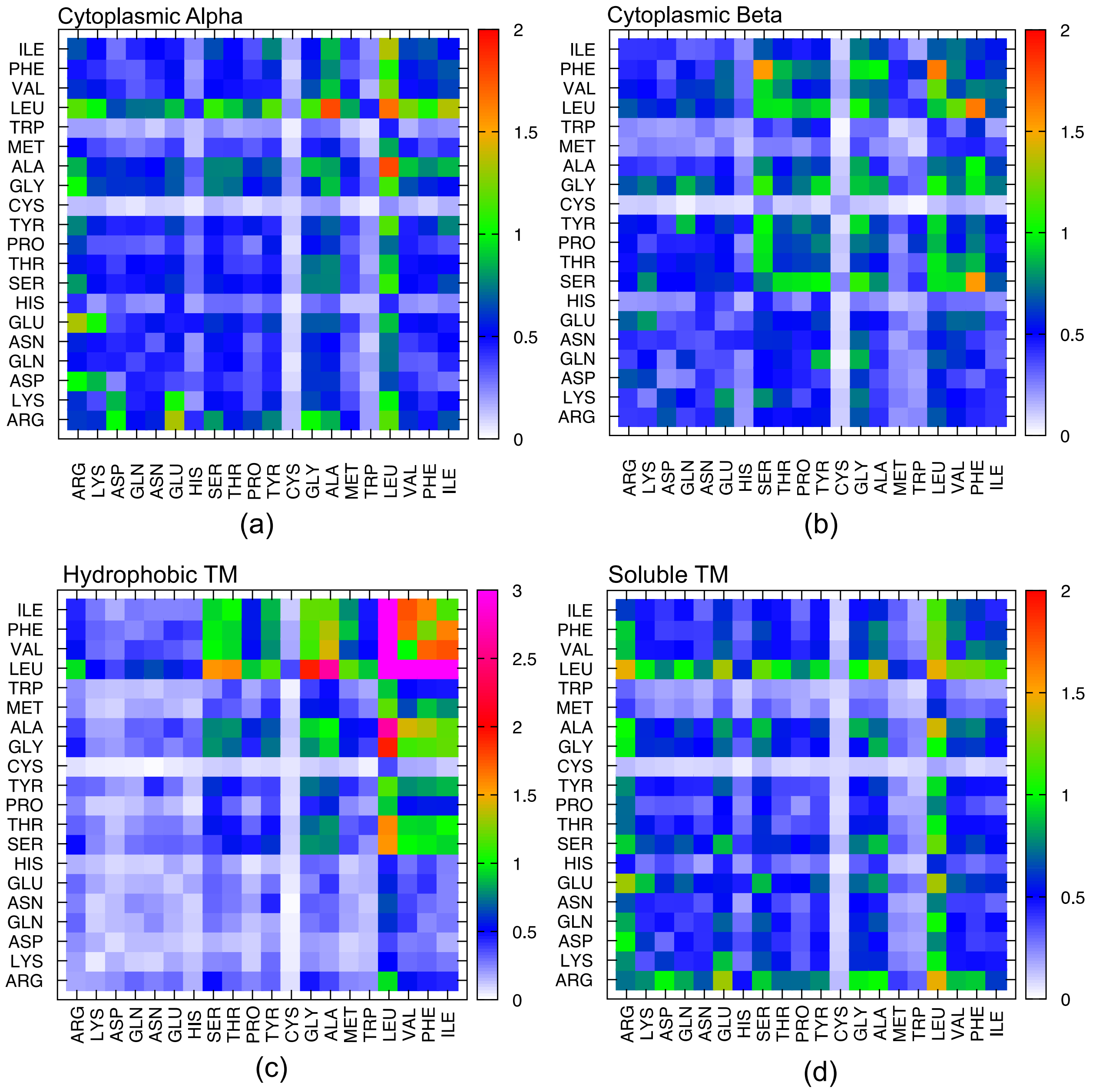

Supplementary Figure 8. Observed frequencies of contacts between residues from two monomers at the interface
